# Origin and Function of Amino Acids in Nectar and Nectaries of *Pitcairnia* Species with Particular Emphasis on Alanine and Glutamine

**DOI:** 10.3390/plants13010023

**Published:** 2023-12-20

**Authors:** Thomas Göttlinger, Gertrud Lohaus

**Affiliations:** Molecular Plant Science and Plant Biochemistry, University of Wuppertal, 42119 Wuppertal, Germany; lohaus@uni-wuppertal.de

**Keywords:** Bromeliaceae, *Pitcairnia*, floral nectar, nectaries, amino acids, alanine aminotransferase, glutamine synthetase

## Abstract

Floral nectar contains sugars and numerous other compounds, including amino acids, but little is known about their function and origin in nectar. Therefore, the amino acid, sugar, and inorganic ion concentrations, as well as the activity of alanine aminotransferase (AlaAT) and glutamine synthetase (GS) in nectar, nectaries, and leaves were analyzed in 30 *Pitcairnia* species. These data were compared with various floral traits, the pollinator type, and the phylogenetic relationships of the species to find possible causes for the high amino acid concentrations in the nectar of some species. The highest concentrations of amino acids (especially alanine) in nectar were found in species with reddish flowers. Furthermore, the concentration of amino acids in nectar and nectaries is determined through analyzing flower color/pollination type rather than phylogenetic relations. This study provides new insights into the origin of amino acids in nectar. The presence of almost all amino acids in nectar is mainly due to their transport in the phloem to the nectaries, with the exception of alanine, which is partially produced in nectaries. In addition, active regulatory mechanisms are required in nectaries that retain most of the amino acids and allow the selective secretion of specific amino acids, such as alanine.

## 1. Introduction

Flowering plants produce floral nectar as a reward to the relevant pollinators, but also to protect from potential herbivores [1,2,3]. The nectar volume and composition as well as the concentration of different compounds varies between plant species. Glucose, fructose, and sucrose are dominant in nectar and the relative amounts of the three sugars are often related to the pollination type of the plant species [4,5] as well as to the color and morphology of the flowers [6]. The nectar composition can also be influenced by other environmental factors, such as light, or water conditions [7,8]. In addition to sugars, nectar contains numerous other compounds, including amino acids, inorganic ions, organic acids, and further secondary compounds, albeit at far lower concentrations than sugars [2,9,10,11].

The total amino acid concentration in nectar is in the micromolar to millimolar range and varies depending on plant species [12,13,14]. The use of nitrogen fertilization also led to an altered or increased amino acid concentration in the nectar [15]. Although all proteinogenic amino acids are commonly found in nectar, the most abundant amino acids are glutamine, asparagine, aspartate, glutamate, alanine, and serine in several plant species [4]. In addition, some non-proteinogenic amino acids were found in nectar, such as β-alanine, γ-aminobutyric acid (GABA), or taurine [16]. Amino acids in nectar are discussed as being adaptations to different types of pollinators [17], either as a source of nitrogen [18], or as phagostimulants, such as phenylalanine for honeybees [15,19]. Proline has also been found in high concentrations in the nectar of some plant species [20]. Honey bees are attracted to proline in nectar [21], and bees can use proline as metabolic fuel in the early flight phase [22]. In the nectar of bat-pollinated flowers, amino acids reduce the ability to distinguish between sugar concentrations [23]. 

In other studies, no connection was found between amino acid concentration in nectar and pollinator type [24,25], and the functional importance of nectar amino acids for pollinators is not clear [2]. In addition, the biological functions of nectar amino acids can vary between plant species [24].

Nectar compounds are produced in so called nectaries, nectar-secreting tissue. In the case of sugars, several metabolic steps are involved, including import of sucrose delivered from the phloem, starch degradation, and sugar synthesis at anthesis [26,27,28,29]. Sucrose or glucose can also be exported from the nectaries through the uniporter Sugar Will Eventually be Exported Transporter (SWEET9) [26]. In addition to this eccrine-based nectar secretion, granulocrine-based nectar secretion has also been proposed for some plant species, based on the ultrastructure of the secretory epithelia [30]. 

Even less is known about the origin of amino acids in nectar or the amino acid metabolism in nectaries [31]. Nectaries are largely supplied by phloem-derived sugars and amino acids [2]. Amino acids can also be produced in the nectaries themselves through the activity of various enzymes, such as glutamine synthetase or alanine aminotransferase [31]. Glutamine synthetase (GS, E.C. 6.3.1.2) catalyzes the synthesis of glutamine from glutamate and ammonium in an ATP-dependent reaction [32], whereas alanine aminotransferase (AlaAT; E.C. 2.6.1.2) catalyzes the reversible conversion of pyruvate and glutamate to alanine and 2-oxoglutarate [33]. However, parallel analyses of nectar and nectaries revealed that the concentration of several amino acids in nectar was lower than in nectaries, suggesting that these amino acids are retained in the nectaries during nectar secretion [34]. Therefore, the question of where nectar amino acids are produced and how they are secreted is still a matter of discussion. In addition, amino acid concentrations in the nectaries can also change under different environmental conditions, for example the concentration of alanine during hypoxic stress in *Cucurbita pepo* [31].

In a previous study with 167 Bromeliaceae species, it was shown that the nectar of several *Pitcairnia* species contained higher amino acid concentrations (more than 10 mM) than the nectar of most other bromeliad species (less than 10 mM). This is the reason why the genus *Pitcairnia* was investigated further in this study. The genus *Pitcairnia* L’Hér. is one of the largest genera of Bromeliaceae, and the species are distributed in Central and South America, mainly in the Andes [35,36]. Most species are mesophytic, grow terrestrially and use C3 photosynthesis [37]. In addition to different nectar compositions, the species also differ in flower color, flower morphology, types of inflorescences, as well as flowering time (day flowering versus night flowering), and these traits are associated with different pollinator types. The pollinators are diverse, mainly hummingbirds and bats, but they are also other bird and insect species [38,39,40,41].

In *Pitcairnia*, as in any other bromeliads, floral nectar is produced by septal nectaries which are located in the basal part of the ovary. They are formed through an incomplete fusion of the carpels [39]. The nectary tissue has a labyrinth-like surface with vascular bundles consisting of phloem and xylem near the parenchyma [29,42]. In general, phloem sap contains sucrose, amino acids, and further compounds [43] which can be transported through the phloem to the nectaries [12]. Water is primarily transported via the xylem, but it has been postulated that the water transport to the flowers occurs primarily in the phloem and only to a lesser extent in the xylem [44]. 

As mentioned above, several models have been developed to illustrate the nectar production of nectaries, with a main focus on sugars, while there are very few studies focusing on amino acid production [31,34]. Therefore, further research is required. The genus *Pitcairnia* is ideal for this, as the flower morphology, the flower color, the type of pollinators, and the amino acid concentration in nectar of different species vary greatly. To gain further insight into the origin and function of amino acids in nectar, the amino acid concentrations in nectar, nectaries, and leaves of numerous *Pitcairnia* species were analyzed in this study. Amino acid concentrations were compared with various floral traits, the pollinator type, and to the phylogenetic relationships of the species to find possible causes for the high amino acid concentrations in the nectar of some *Pitcairnia* species. In addition, the origin of amino acids in nectar and the synthesis of the most common amino acids alanine and glutamine were examined in more detail.

## 2. Materials and Methods

### 2.1. Origin of Plant Material

*Pitcairnia* plants grown in tropical glasshouses in the Botanical Garden and Botanical Museum Berlin (Germany), the Zoological–Botanical Garden Stuttgart (Germany) and the Botanical Garden of the University of Heidelberg (Germany) were used for the experiments (Supplementary Table S1). 

### 2.2. Characteristics of the Pitcairnia Species

The selected *Pitcairnia* species grew terrestrial and utilized C3 photosynthesis, but they differed in the type of inflorescence, flower morphology, color of their bracts, sepals or petals, and mode of pollination. Most flowers are long (between 4 and 10 cm) and narrow. The species showed three types of inflorescences (Supplementary Figure S1): raceme inflorescence (each flower has a short, unbranched peduncle along the shoot; Supplementary Figure S1A), spike inflorescence (the flowers are located directly at the central axis of the inflorescence, Supplementary Figure S1B), and panicle inflorescence (the peduncles are branched and bear multiple flowers, Supplementary Figure S1C). Of the 30 species, 28 species are supposedly pollinated mainly by hummingbirds (trochilophilous), and two species mainly by bats (chiropterophilous) (Supplementary Table S1).

### 2.3. Sampling of Plant Material

The plant material (leaves, nectaries, and nectar) was harvested shortly after anthesis.

Nectar samples of 30 *Pitcairnia* species (Supplementary Table S1) were collected from each flower using a micropipette [4]. The nectar was outside the ovary, and between 10 and 50 µL of nectar could be collected per flower. To detect possible pollen contamination, nectar samples were examined microscopically for the presence of pollen.

The septal nectary of each flower from 13 *Pitcairnia* species was dissected from the ovaries using a scalpel and a stereomicroscope [34,42]. The nectaries were rinsed with ultrapure water to remove any nectar that might be present. Due to the different sizes of the nectaries in the different species and the small amount of nectary tissue, nectary tissue from 5 to 15 flowers had to be pooled to obtain the required amount for analysis (25 mg tissue per sample).

Leaf samples of 30 *Pitcairnia* species were taken with a razor blade.

Three samples per tissue (leaves, nectaries) and nectar of three different plants were collected from each *Pitcairnia* species. For storage, samples were frozen in liquid nitrogen and stored at −80 °C until analysis of sugars, amino acids, and inorganic ions was performed.

### 2.4. Extraction of Organic Metabolites and Inorganic Ions from Plant Tissue

Soluble metabolites (sugars, amino acids, organic acids) and inorganic ions were extracted from nectaries or leaves using chloroform-methanol-water extraction. Therefore, between 150 and 200 mg of leaf tissue or 50 mg of nectaries were ground into a fine powder in liquid nitrogen. To this powder, 5 mL chloroform/methanol (3:7, *v*/*v*) mixture was added. The samples were homogenized and incubated on ice for 30 min. The homogenate was then extracted twice with 3 mL water. The aqueous phases of two extractions were combined and evaporated in a rotary evaporator. Afterwards, the dried residue was dissolved with either 1 mL (leaves) or 0.5 mL (nectaries) of ultrapure water (Millipore, Burlington, MA, USA). After syringe filtration (0.45 μm cellulose-acetate; Schleicher and Schuell, Dassel, Germany), the samples were stored at −80 °C until analysis.

### 2.5. Analysis of Sugars, Amino Acids, Inorganic Ions, and Organic Acids in Nectar, Nectaries, and Leaves

The nectar samples and the extracts of nectaries and leaves were analyzed for the concentrations of sugars, amino acids, inorganic ions, and organic acids using different HPLC systems [4]:

The concentrations of sugars in nectar, nectaries, and leaves were determined separately via HPLC (Thermo Fisher Scientific Dionex ICS-5000 HPIC System; Thermo Fisher Scientific, Waltham, MA, USA). The sugars were eluted isocratically using an anion exchange column (Dionex CarboPac^TM^ PA10 4 × 250 mm; Thermo Fisher Scientific, Waltham, MA, USA) and detected with a pulse amperometric detector.

The analyses of amino acids were performed via HPLC (Thermo Fisher Scientific UltiMate 3000; Thermo Fisher Scientific, Waltham, MA, USA). The concentration of free amino acids (alanine, arginine, aspartate, asparagine, glutamate, glutamine, glycine, histidine, isoleucine, leucine, lysine, methionine, phenylalanine, proline, serine, threonine, tryptophan, tyrosine, valine) and additionally β-alanine, γ-aminobutyric acid (GABA) and taurine could be detected in the different plant materials with a fluorescence detector after separation on a reversed phase column (Merck LiChroCART^®^ 125-4 using Superspher^®^ 100 RP-18 endcapped; Merck, Darmstadt, Germany).

Inorganic ions (anions: chloride, nitrate, phosphate, sulfate; cations: potassium, sodium, magnesium, calcium) and organic acids (pyruvate, malate) were detected using their electronic conductivity. For the analysis of anions (inorganic anions and organic acids), an anion exchange column (Dionex IonPac^TM^ AS11 4 × 250 mm; Thermo Fisher Scientific, Waltham, MA, USA) was used; for the analysis of cations, a cation exchange column (Dionex CS 12A, 4 × 250 mm; Thermo Fisher Scientific, Waltham, MA, USA) was used.

The chromatograms were evaluated with an integration program (Peaknet 5.1; Dionex Corp, Sunnyvale, CA, USA; Chromeleon 7.2; Thermo Fisher Scientific, Waltham, MA, USA). The concentrations of the metabolites, inorganic ions, and organic acids in the samples were determined using calibration curves for each component. The respective concentrations in the extracts of leaves and nectaries are given in µmol g^−1^ fresh weight (FW). To express the concentration in these tissues in millimolar (mM), the water content of leaf cells (86%) and nectary cells (75%) was used [45].

### 2.6. Analysis of Starch in Nectary Tissue and Leaf Tissue

According to a modified protocol from Riens et al. [46], the content of starch was determined in nectaries and leaves. From the chloroform-methanol-water extraction (see Section 2.4), the lower phase containing chloroform was washed with 5 mL 96% ethanol, centrifuged and the supernatant was discarded. The resulting pellet was resuspended with 2 mL KOH and incubated at 80 °C for 3 h, and then 350 µL acetic acid was added. The starch was dissolved in acetate buffer and was cleaved enzymatically using α-amylase and amyloglucosidase. The resulting glucose was determined using an enzymatic assay with hexokinase/glucose-6P dehydrogenase.

### 2.7. Analysis of the Enzyme Activity of Glutamine Synthetase in Leaves and Nectaries

In leaves and nectaries, the activity of glutamine synthetase was analyzed according to a modified protocol from [47]. Leaves (200–300 mg) and nectaries (50–100 mg) were milled to a fine powder in liquid nitrogen. The milled tissue was mixed gently with 1 mL (leaves) or 0.5 mL (nectaries) extraction buffer containing 50 mM Hepes-KOH (pH 7.7), 10% (*v*/*v*) glycerin, 5 mM MgCl_2_, 1 mM EDTA, and 0.1% (*v*/*v*) Triton X-100. A total of 280 µL extract was added to 400 µL assay buffer containing 10 mM hydroxylamine, 10 mM MgCl_2_, 50 mM glutamate, and 10 mM ATP. Further, normalization of enzyme activity followed, using control assays without ATP added. The sample preparation was stored for 30 min at 37 °C. After that, 400 µL staining mixture was added to each assay, and these were centrifugated for 2 min at room temperature. The extinction of the supernatant was determined photometrically at 535 nm. The amounts of γ-glutamyl hydroxamate could be determined using calibration curves measured in parallel from 0 to 1.2 µmol.

### 2.8. Analysis of the Enzyme Activity of Alanine Aminotransferase in Leaves and Nectaries

According to a modified protocol from Solhaug et al. [31], the enzyme activity of alanine aminotransferase in leaves and nectaries was quantified. Briefly, leaves (200–300 mg) or nectaries (50–100 mg) were milled in liquid nitrogen, and then blended in a ratio of 1 to 3 with extraction buffer containing 100 mM Tris-HCl (pH 7.5), 5 mM EDTA, and 1 mM DTT, followed by centrifugation at 6000× *g* for 15 min at 4 °C. An amount of 100 µL of the supernatants was mixed with 900 µL of the assay buffer containing 100 mM Tris-HCl (pH 8.0), 10 mM α-ketoglutarate, 70 mM alanine, 0.28 mM NADH, and 1.2 U mL^−1^ lactate dehydrogenase (LDH). The assay mixture was incubated for 30 min at 22 °C. Finally, the degradation of NADH was measured at an absorbance of 340 nm. In addition, normalization of enzyme activity followed using control assays without LDH and alanine added. The results were evaluated with a standard curve of NADH (0–1000 µM) to obtain the unit for the enzyme activity: µmol g^−1^ FW min^−1^.

### 2.9. Influence of the Growth Site (Different Botanical Gardens) on Amino Acid Concentration in Nectar

Since the nectar samples of the *Pitcairnia* species were collected in different botanical gardens (Supplementary Table S1), it was tested whether this had an influence on the nectar composition. Therefore, the amino acid concentrations of the same *Pitcairnia* species from different botanical gardens were analyzed (Supplementary Figure S2). The amino acid concentrations in nectar of *P. imbricata* (Supplementary Figure S2A) and *P. sceptrigera* (Supplementary Figure S2B) from the botanical gardens of Berlin and Stuttgart as well as Berlin and Heidelberg showed no significant differences (*p* < 0.05). This has already been shown in a previous work for sugars in nectar [4].

### 2.10. Phylogenetic Analysis

For the analyzed *Pitcairnia* species, a simplified phylogenetic tree was constructed considering the molecular analyses from Schubert [48] and Saraiva et al. [41]. Mesquite (version 3.81, www.mesquiteproject.org, accessed on 17 March 2023) is a modular system for evolutionary analysis and was used to create the schematic tree. Flower color, inflorescence, and concentrations of metabolites and inorganic ions in nectaries and nectar were mapped to the species level in the phylogenetic tree to demonstrate variation within the genus *Pitcairnia*.

Comparative methods were performed to verify the effect of phylogeny or shared ancestry of the species. Therefore, to validate the correlation of the presence or absence of traits, BayesTraits (Version 4.0.1, www.evolution.rdg.ac.uk, accessed on 20 March 2023) was used. Each feature has to be simplified into only two groups. The flower color feature was divided into the groups “red” (reddish flowers and reddish bracts) and “not red” (yellow/white and greenish/white flowers), and the amino acid concentration feature was divided into “low concentration” (<10 mM) and “high concentration” (>10 mM). Using this, a discrete, dependent and independent model were performed to analyze the methods against each other to determine a likelihood ratio [49]. The Chi-square distribution proved the significance (*p* < 0.05) for the results.

### 2.11. Statistical Analysis

Phylogenetic Generalized Least Squares (PGLS) regression analysis was conducted to examine whether the phylogenetic relationship influences similarity in species traits. For this calculation, the four color groups (reddish sepals and petals, reddish bracts, yellow/white sepals and petals, greenish/white sepals and petals) and the actual values of the amino acid concentrations were used. The PGLS analysis was performed using the “caper” package [50] in R (version 4.2.2, www.r-project.org, accessed on 9 March 2023).

The normal distribution of the samples was confirmed using quantile–quantile plots. When examining metabolite and inorganic ion concentrations, comparison of two groups was performed applying *t*-tests, and comparisons of more than two groups were performed through applying one-way ANOVAs, followed by Tuckey post hoc test to determine significant differences.

For Pearson’s rank correlation, the ‘cor’ function of the ‘corrplot’ package in R was applied [51].

The data of the *Pitcairnia* species studied were divided into different groups with respect to different types of inflorescence or flower traits. A possible influence of these groups on the metabolites and inorganic ions was examined through applying a Principal Component Analysis (PCA) [4]. For a balanced system within the PCA, the groups were formed with equal sample numbers. Based on inflorescence, the data of the *Pitcairnia* species were divided into three groups (raceme inflorescence: *P. bromeliifolia*, *P. corallina*, *P. chiapensis*; spike inflorescence: *P. spicata*, *P. flagellaris*, *P. sceptrigera*; panicle inflorescence: *P. chiriquensis*, *P. echinata var. vallensis*, *P. utcubambensis*) with three species each. Based on flower length, three groups (up to 4 cm: *P. atrorubens*, *P. chiapensis*, *P. corallina*, *P. integrifolia*, *P. utcubambensis*; from 4 to 6 cm: *P. altensteinii var. altensteinii*, *P. angustifolia*, *P. bromeliifolia*, *P. flagellaris*, *P. spicata*; above 6 cm: *P. albolutea*, *P. capixaba*, *P. carnosepala*, *P. nigra var. nigra*, *P. poeppigiana*) were also built, with each group containing the data of five species. Due to the many color combinations of the flower leaves (sepals, petals) and bracts, the data of the species were divided into two groups (reddish flowers and bracts: *P. angustifolia*, *P. poeppigiana*, *P. imbricata*, *P. maidifolia*; yellow/white and greenish/white flowers: *P. capixaba*, *P. longissimiflora*, *P. recurvata*, *P. sceptigera*), each with four species per group. In addition, Permutional Multivariate Analysis of Variance (PERMANOVA) was applied to determine the relative importance of different types of inflorescence or flower traits on the metabolites and ions in leaves, nectaries, and nectar [52]. 

## 3. Results

### 3.1. Influence of Inflorescence Type, Flower Length, and Flower Color

**Figure 1:**
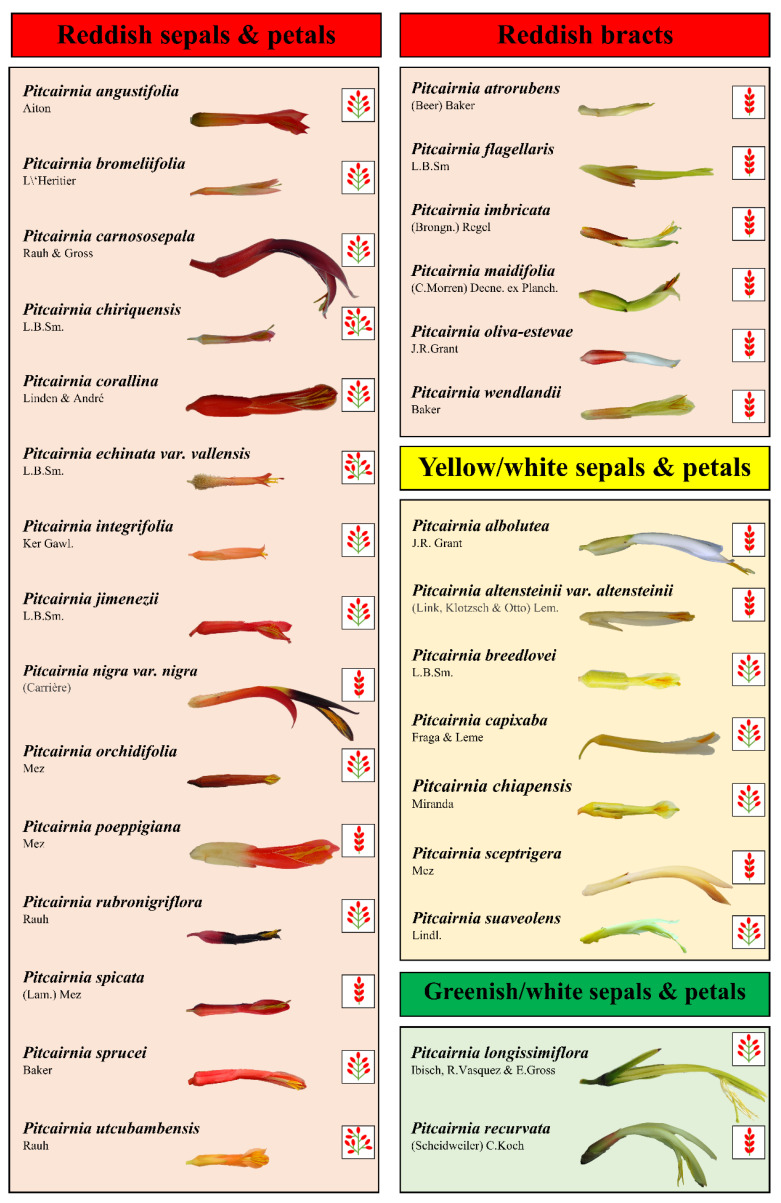
The analyzed *Pitcairnia* species were separated using four flower-color groups. Thereby, the four groups include different number of species. Reddish sepals and petals: 15 species; reddish bracts: 6 species; yellow/white sepals and petals: 7 species; greenish/white sepals and petals: 2 species. For each species the inflorescence type was shown, as well as raceme, spike, and panicle (images of the inflorescence type can also be found in Supplementary Figure S1).

**Figure 2:**
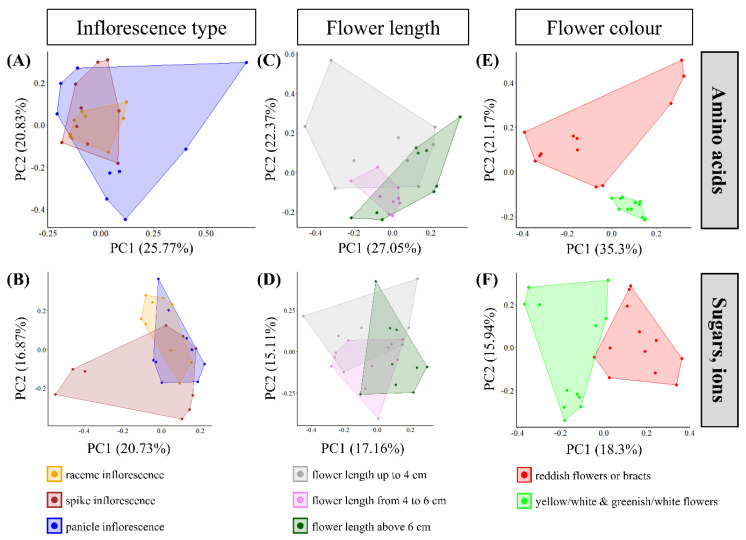
Scatterplots of Principal Component Analysis (PCA) in rotated space of different inflorescence types ((**A**,**B**); raceme, spike, panicle inflorescence), of different flower length ((**C**,**D**); flower length up to 4 cm, from 4 to 6 cm, above 6 cm), and of different flower color ((**E**,**F**); reddish flowers or bracts, yellow/white, and greenish/white flowers). Amino acid data (**A**,**C**,**E**) and, separately, sugar and inorganic ion data (**B**,**D**,**F**) in leaves, nectaries, and nectar were used for PCA.

Figure 1 shows the 30 *Pitcairnia* species with three types of inflorescences (raceme, spike, panicle; Supplementary Figure S1), different flower lengths (4–10 cm), and different colors for sepals, petals, and bracts (red, white, yellow, or green; Figure 1 and Table S1). To investigate whether the differences in amino acid, sugar, and inorganic ion concentrations in leaves, nectaries, and nectar of these species can be explained using the different types of inflorescences or flower traits, PCAs were performed (Figure 2). For the PCAs, either the amino acid concentrations in leaves, nectaries, and nectar (Figure 2A,C,E) or the concentrations of sugars and inorganic ions (Figure 2B,D,F) were used.

PCA performed with amino acid data explained 46.6% (Figure 2A) and with sugar and inorganic ion data 37.6% (Figure 2B) of the total variance of the data based on principal components subdivided using inflorescence type. For both PCAs performed with amino acid data or with sugar and inorganic ion data, the graphical evaluation cannot be separated on the basis of the three types of inflorescence. 

Considering the data grouped using flower length, no separated clustering of the grouped *Pitcairnia* species can be detected either (Figure 2C,D). As before, the PCA conducted with amino acid data explained 44.4% of the total variance with the two principal components (Figure 2C), whereas PCA performed with sugar and inorganic ion data explained only 32.3% (Figure 2D).

Considering amino acid data of leaves, nectaries, and nectar in the PCA, a separation can be determined based on the flower color. Thereby, the species with reddish and the species with yellow/white or greenish/white flower color are clustered together (Figure 2E). The two principal components explained 56.5% of the total variance (Figure 2E). The PCA conducted with sugar and inorganic ion data (Figure 2F) showed less segregation of the flower groups than the PCA carried out with amino acid data. This is also reflected in the two principal components which explained only 34.2% of total variance. 

The PERMANOVA supports the graphical evaluation of the PCA. Considering sugar and inorganic ion data, the flower color explains only 26% of the data variations in nectar, nectaries, and leaves (Supplementary Table S2B; *p* ≤ 0.001), whereas the PERMANOVA with amino acid data explains 58% of the data variation with the flower color (Supplementary Table S2A; *p* ≤ 0.001). Due to this, some individual amino acids were also investigated with regard to their influence on data variation in connection with flower color using PERMANOVA. Alanine stands out particularly in this analysis, since the PERMANOVA performed with alanine explained 82% of the data variation with the flower color (Supplementary Table S2C).

### 3.2. Sugar, Amino Acid, Inorganic Ion Concentration in Leaves, Nectaries, and Nectar in the Different Flower-Color Groups

Since flower color appears to have the highest influence on nectar composition, the species were further divided into four color groups (Figure 1 and Supplementary Table S1). These groups also take pollinator types into account. The “reddish group” was divided into two groups, one group included 15 species with reddish sepals and petals, the second group included six species with reddish bracts, while the sepals and petals were white or yellowish. The “non-reddish group” was also divided in two groups. One group included seven species with yellow/white sepals and petals, and the other group included only two species with greenish/white sepals and petals. The latter group is related to the pollination of these species by bats, while the *Pitcairnia* species of the other three groups are pollinated primarily by hummingbirds and probably by other birds or insects.

**Figure 3:**
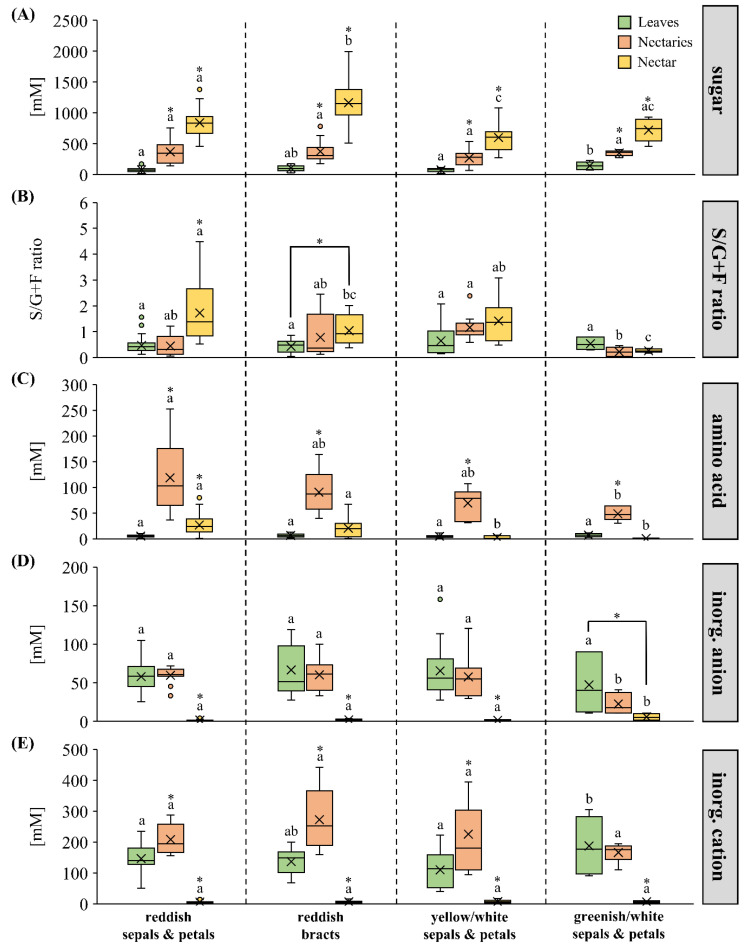
Sum of sugars (glucose, fructose, sucrose), sucrose-to-hexoses ratio (S/G+F), sum of amino acids, sum of inorganic anions, and cations in leaves, nectaries, and nectar (**A**–**E**). The metabolites and ions are separated by four flower-color groups in the boxplot diagrams (reddish sepals and petals; reddish bracts; yellow/white sepals and petals, greenish/white sepals and petals). The shown data for leaves and nectar include 30 *Pitcairnia* species and nectaries includes 13 species (n = 3). Different letters represent significant differences in metabolites or inorganic ions, respectively, between the four flower-color groups (Tukey’s HSD; *p* < 0.05). The asterisks show significant differences in the flower-color groups, respectively, between leaves, nectaries, or nectar (*p* < 0.05).

The sugar, amino acid, and inorganic ion concentrations in leaves, nectar, and nectaries of the different species in the four color groups are shown in Figure 3. In all groups, the concentration of the sum of sugars (glucose, fructose, sucrose) was lowest in leaves (10–200 mM), followed by nectaries (70–800 mM), and highest in nectar (300–2000 mM) (Figure 3A). A comparison of the four color groups showed that the sugar concentrations in the nectaries were similar in all groups, whereas in the nectar, the sugar concentration was higher in the reddish groups than in the groups with yellow/white or greenish/white sepals and petals.

Also, in *Pitcairnia* species with reddish sepals and petals, the sucrose-to-hexoses ratio in nectar was higher than in nectaries, whereas in the other groups the ratios in nectaries and nectar were similar (Figure 3B). Nectar of species with greenish/white sepals and petals showed a lower sucrose-to-hexoses ratio than nectar from species in the other three groups. This is also reflected in the corresponding sugar ratios in the nectaries. 

In leaves, nectaries and nectar, 19 proteinogenic amino acids (alanine, arginine, aspartate, asparagine, glutamate, glutamine, glycine, histidine, isoleucine, leucine, lysine, methionine, phenylalanine, proline, serine, threonine, tryptophan, tyrosine, valine) and three non-proteinogenic amino acids (β-alanine, γ-aminobutyric acid/GABA, taurine) were detected in different concentrations. In all groups, the total amino acid concentration was higher in the nectaries than in leaves or nectar (Figure 3C). A comparison of the four color groups showed that the total amino acid concentrations in the nectaries and nectar were higher in the groups with reddish sepals and petals or bracts than in the groups with yellow/white or greenish/white flowers (*p* < 0.05). 

In all groups, the concentration of inorganic anions (chloride, nitrate, sulfate, phosphate) was similar in leaves and nectaries, whereas it was much lower in nectar (Figure 3D). A comparison of the four color groups showed that the concentration of inorganic anions in nectar was higher in the group with greenish/white sepals and petals than in the other groups (*p* < 0.05).

With the exception of the species with greenish/white flowers, the concentration of inorganic cations (potassium, sodium, magnesium, calcium) was highest in nectaries, followed by leaves and much lower in nectar (Figure 3E). No difference was found in the concentration of inorganic cations in the nectar of the four groups (*p* < 0.05).

Comparison of the ratios of the various compounds (sum of sugars to sum of amino acids or sum of inorganic ions) revealed only minor differences in the leaves and nectaries of the four groups (Supplementary Table S3). Different ratios were only found in the nectar of the different groups of *Pitcairnia* species. In nectar, the ratio of the sum of sugars to the sum of amino acids was between 82 and 244 in the two reddish flower groups and between 760 and 1080 in the groups with yellow/white or greenish/white sepals and petals. This means that the nectar of species in the reddish groups contained many more amino acids in relation to sugars than the nectar of the other species. In contrast, the ratio sum of sugars to the sum of inorganic ions was higher in the two reddish flower groups (166–213) than in the groups with yellow/white or greenish/white sepals or petals (80–100).

### 3.3. Comparison of Different Amino Acids in Leaves, Nectaries, and Nectar

**Figure 4:**
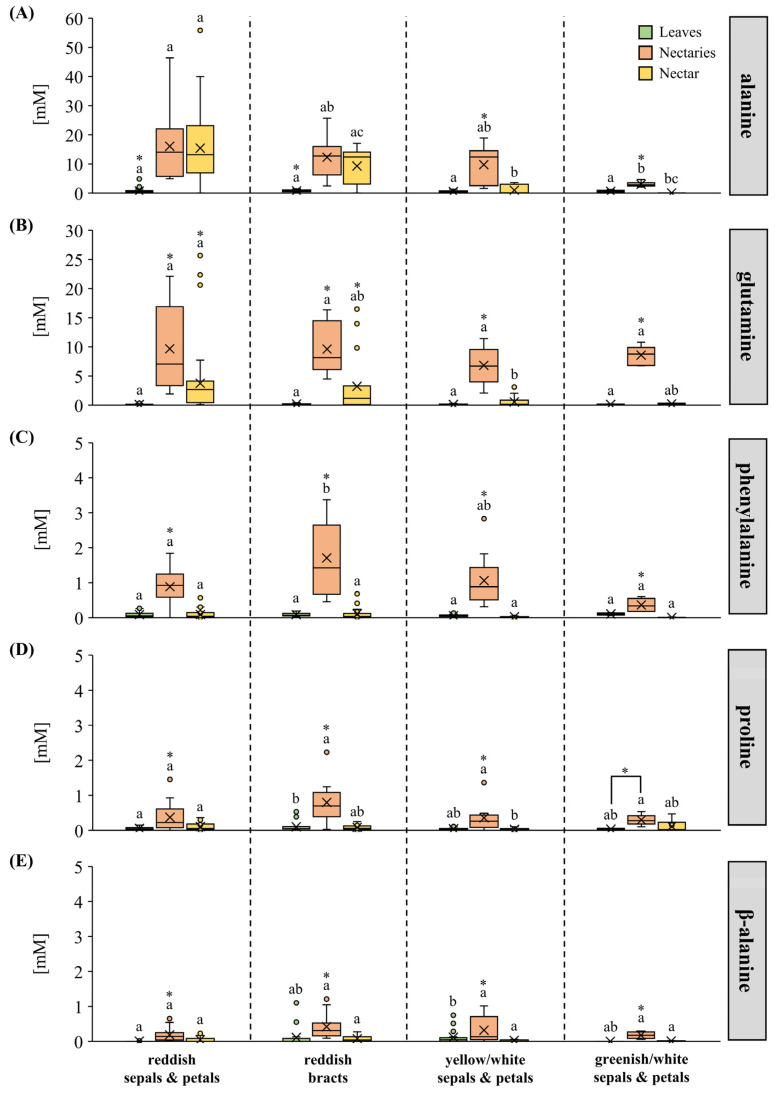
The concentrations of alanine (**A**), glutamine (**B**), phenylalanine (**C**), proline (**D**), and β-alanine (**E**) in leaves, nectaries, and nectar. Each amino acid is separated by the four flower-color groups in the boxplot diagrams (reddish sepals and petals; reddish bracts; yellow/white sepals and petals, greenish/white sepals, and petals). The shown data for leaves and nectar includes 30 *Pitcairnia* species, and nectaries includes 13 species (n = 3). Different letters represent significant differences in metabolites or inorganic ions, respectively, between the four flower-color groups (Tukey’s HSD; *p* < 0.05). The asterisks show significant differences in the flower-color groups, respectively, between leaves, nectaries, or nectar (*p* < 0.05).

As total amino acid concentrations in both nectaries and nectar varied between *Pitcairnia* species, the amino acid composition and the concentration of individual amino acids were analyzed in more detail (Table 1, Figure 4). Alanine was the most abundant amino acid in nectar of almost all *Pitcairnia* species with reddish sepals and petals or bracts (Table 1). In species with yellow/white flowers, the main amino acid varied from species to species; in some species it was alanine, and in other species it was glutamine or asparagine (Table 1). Nectar of species with greenish/white flowers was dominated by amides, glutamine, and asparagine.

In addition to the high alanine concentrations in the nectar of *Pitcairnia* species with reddish flowers or bracts, high alanine concentrations in nectaries were also found (Figure 4A). Species with reddish flowers or bracts showed similar alanine concentrations in nectar and nectaries, while species from both other color groups had lower concentrations in nectar than in nectaries (Figure 4A). The glutamine concentration in the nectaries of the four groups was similar, and the concentration was consistently higher than that in the nectar (Figure 4B). The concentrations of phenylalanine (Figure 4C), proline (Figure 4D), and β-alanine (Figure 4E) were low in nectaries and nectar and the differences between the four color groups were only small (Figure 4C–E). In the leaves, there were hardly any differences between the four color groups for alanine, glutamine, phenylalanine, proline, or β-alanine (Figure 4A–E). 

### 3.4. Activities of Alanine Aminotransferase and Glutamine Synthetase

The most abundant amino acids in the nectar of *Pitcairnia* species were alanine and glutamine (Figure 4A,B). Therefore, the activity of alanine aminotransferases (AlaAT) and glutamine synthetases (GS) were measured in leaves, nectaries, and nectar, whereby no activity was detectable in the nectar. Since large amounts of nectary material were required for enzymatic studies, measurements were only possible for nine *Pitcairnia* species. In order to have enough *Pitcairnia* species per group, the flower-color groups were reduced to two, a group with reddish sepals, petals, or bracts (six species) and a group with yellow/white or greenish/white sepals and petals (three species).

**Figure 5:**
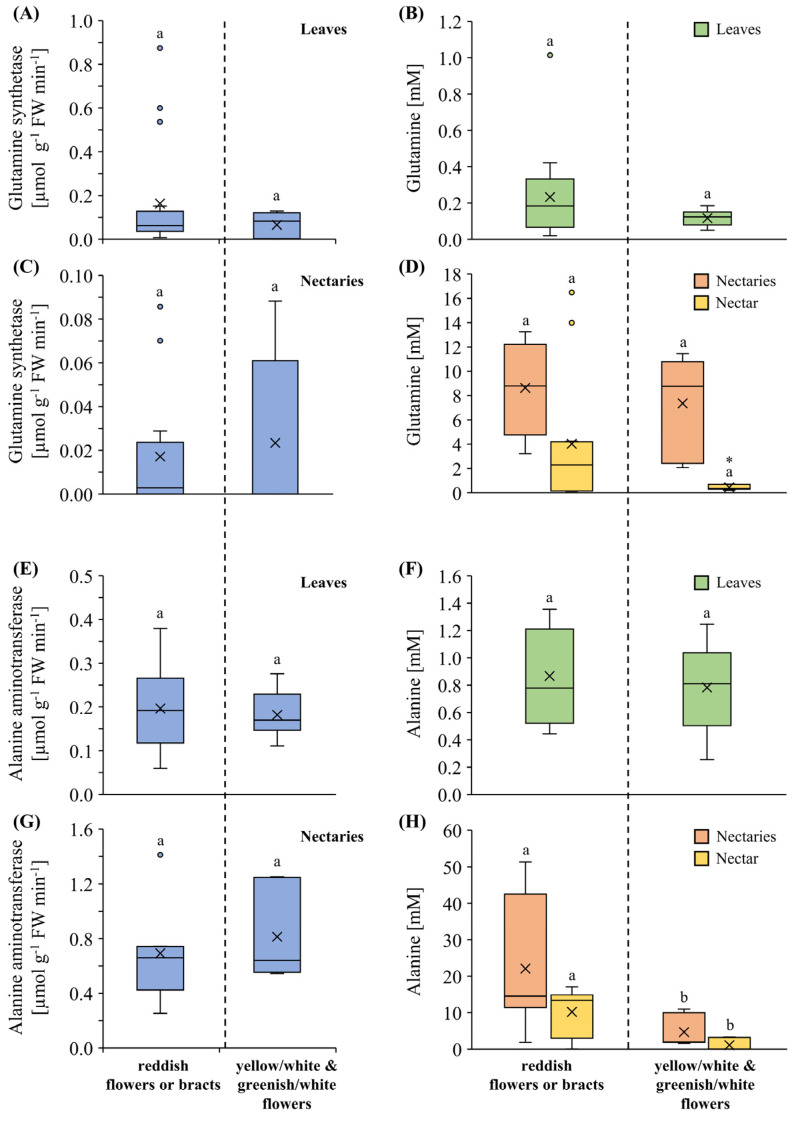
The enzyme activity (µmol g^−1^ FW min^−1^) of glutamine synthetase is shown in leaves (**A**) and nectaries (**C**). Glutamine concentration in leaves (**B**), as well as in nectaries and nectar (**D**), is shown for comparison. Further, the enzyme activity of alanine aminotransferase is presented in leaves (**E**) and nectaries (**G**). Alanine concentration in leaves (**F**), as well as in nectaries and nectar (**H**), is shown for comparison. For both enzymatic studies, the same nine *Pitcairnia* species were analyzed in each case. The enzyme activity and associated amino acid concentration is separated by two flower-color groups (reddish flowers or bracts: *P. angustifolia*, *P. imbricata*, *P. maidifolia*, *P. oliva-estavae*, *P. poeppigiana*, *P. wendlandii*; yellow/white and greenish/white flowers: *P. capixaba*, *P. longissimiflora*, *P. sceptrigera*). Different letters represent significant differences in enzyme activity or amino acid concentration, respectively, between the two flower-color groups (Tukey’s HSD; *p* < 0.05). The asterisks show significant differences in the flower-color groups, respectively, between nectaries or nectar (*p* < 0.05).

In general, the activity of GS was higher in leaves than in nectaries (Figure 5A,C), whereas, conversely, the activity of AlaAT was higher in nectaries than in leaves (Figure 5E,G).

In the leaves of both the reddish and the yellow/white or greenish/white group, the GS activity was similar (Figure 5A), and the same applies to the glutamine content in leaves of the same species in the two groups (Figure 5B). The GS activity in the nectaries was about 5-fold lower than in leaves, and there was no significant difference between the two flower-color groups (Figure 5C). The glutamine content in nectaries or nectar of the same species in the two groups did not show any differences either (Figure 5D).

The AlaAT activity was similar in leaves of species with reddish or yellow/white and greenish/white flowers (Figure 5E). The alanine content in leaves of the same species in the two groups did not show any difference either (Figure 5F). The AlaAT activity in nectaries was generally about four times higher than in leaves, but, again, no significant difference was found between species of both groups (Figure 5G). In contrast, the alanine concentration in nectaries and in the nectar of the reddish flower group was significantly higher than in species of the yellow/white and greenish/white group (Figure 5H). 

### 3.5. Pyruvate and Malate Concentrations and Their Relationship to Alanine Synthesis

**Figure 6:**
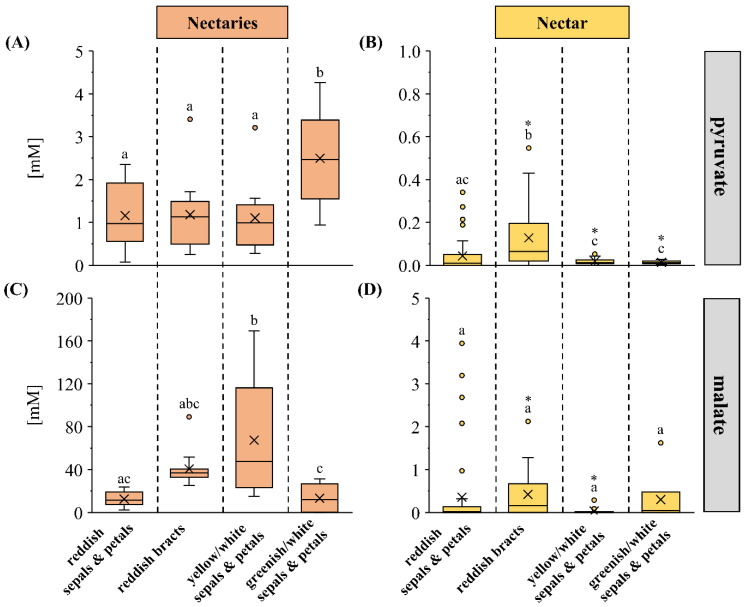
The concentrations of pyruvate (**A**,**B**) and malate (**C**,**D**) are shown in nectaries (**A**,**C**) and nectar (**B**,**D**). Each organic acid is separated by four flower-color groups in the boxplot diagrams (reddish sepals and petals; reddish bracts; yellow/white sepals and petals, greenish/white sepals and petals). The shown data for nectar includes 30 *Pitcairnia* species and nectaries includes 13 species (n = 3). Different letters represent significant differences in organic acids, respectively, between the four flower-color groups (Tukey’s HSD; *p* < 0.05). The asterisks show significant differences in the flower-color groups, respectively, between nectaries or nectar (*p* < 0.05).

The concentration of pyruvate, a precursor for the alanine synthesis, was measured in nectaries and nectar from *Pitcairnia* species of the four flower-color groups. The mean pyruvate concentrations in the nectaries varied between 1 and 3 mM in the different flower-color groups (Figure 6A), with the mean concentrations in nectaries of species with greenish/white sepals and petals being highest and differing significantly from the other three flower-color groups (Figure 6A; *p* < 0.05). Pyruvate was hardly detectable in the nectar, since the concentration was usually below 0.05 mM. Only species with reddish bracts showed slightly higher pyruvate concentration (Figure 6B; *p* < 0.05).

As malate is one of the most common organic acids in plant cells and pyruvate can be synthesized from it, the concentrations of malate were also analyzed. The mean malate concentrations in nectaries ranged from 15 to 70 mM, whereas in nectar, the mean concentrations ranged from only 0.1 to 0.5 mM (Figure 6C,D). *Pitcairnia* species with yellow/white sepals and petals showed significantly higher malate concentrations in nectaries than the species of the other-color flower groups (*p* < 0.05). The mean malate concentrations in nectar were less than 0.5 mM, and no significant differences were found between the four color groups (Figure 6D). 

### 3.6. Phylogenetic Distribution Associated with Flower Color and Nectar Amino Acids

**Figure 7:**
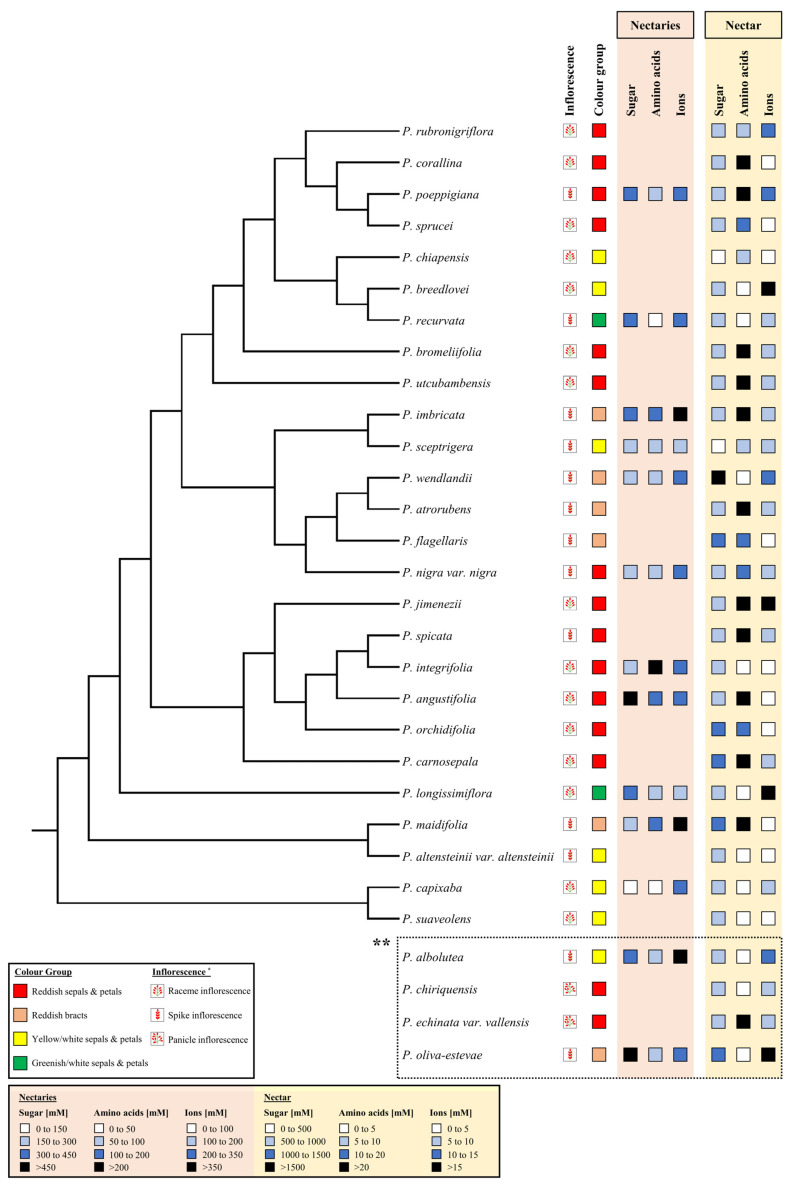
Simplified phylogenetic tree of all analyzed *Pitcairnia* species combining molecular and morphological findings, which are based on two phylogenetic investigations [41,48]. The phylogenetic tree was created using Mesquite 3.81. * Supplementary Figure S1. ** No molecular phylogenetic data are available.

Of the 30 *Pitcairnia* species used for sugar, amino acid, and inorganic ion analyses, 26 species could be used to construct a schematic and simplified phylogenetic tree (Figure 7). Comparative analysis with BayesTraits was used based on this tree to determine whether the amino acid concentrations of the species within this genus arose independently of common ancestry. In this context, the presence and absence of the reddish color in the flowers and bracts was compared to the concentrations of amino acids in nectar. For simplification, the analyzed traits had to be divided into two groups: “red” (reddish flowers and bracts) and “not red” (yellow/white and greenish/white flowers), and the amino acid concentration was divided into “low concentration” (<10 mM) and “high concentration” (>10 mM). In this simplified analysis, a likelihood ratio of 18.81 could be calculated for amino acids (*p* ≤ 0.001), which suggests rejection of the hypothesis that flower color and high amino acid concentrations evolved independently. Instead, the comparative analysis gives strong evidence that the presence of reddish color in flowers (sepals, petals) or bracts and a high content of amino acids in nectar (>10 mM) evolved together.

In a more detailed analysis, a phylogenetic correlation using PGLS to determine the factor lambda was performed in order to examine the extent to which the phylogeny influences the individual traits. For this calculation, instead of the simplified two, all four color groups were used (reddish sepals and petals, reddish bracts, yellow/white sepals and petals, greenish/white sepals and petals). Whereas in comparative analysis with BayesTraits, only the presence of high amino acid concentrations was considered; for the PGLS, we considered the actual concentrations of amino acids in nectar. The estimated lambda value of the amino acids in connection with the four color groups is exactly 0 (*p* ≤ 0.001), whereby the observed variations indicate that patterns of trait similarity amongst species are independent of phylogeny. This is also evident considering the fact that differences in amino acid composition and concentration in nectar have been found in closely related species, for example in *P. wendlandii* and *P. atrorubens* or in *P. spicata* and *P. integrifolia* (Figure 7). However, there are also individual examples of similar amino acid concentrations in nectar from related species, for example in *P. capixaba* and *P. suaveolens*. But, overall, the differences in the amino acid concentration in the nectar of closely related species predominate (Figure 7).

## 4. Discussion

### 4.1. Relationship between Amino Acids in Nectar, Flower Color, and Pollinator Type 

Plant–pollinator interaction is influenced by a variety of floral characteristics, such as flowering time, flower morphology, color, scent, nectar availability, as well as nectar composition [53]. For bromeliads and other plant groups, it has been shown that the sugar composition in the nectar of different species correlates with the pollinator type [4,5,54,55,56]. As for the amino acids in nectar, the role is not yet fully understood [2]. A common assumption is that the amino acid composition also correlates with the preferences of certain pollinators [15,17,24]. The amino acids do not necessarily have to serve as a source of nitrogen, they can also increase the taste, and thus the attractiveness, of the nectar, such as proline [20,57,58]. Proline in nectar also attracts hummingbirds [59] in addition to bees [21]. However, the concentration of proline in the nectar of all *Pitcairnia* species was low, and there were no differences between the flower-color groups (Figure 4D). Phenylalanine, which was present at high concentrations in the nectar of several bee-pollinated plant species [15], showed only very low concentrations in the nectar of the analyzed *Pitcairnia* species (Figure 4C). This means that these two amino acids probably do not play an important role in the nectar of *Pitcairnia* species. 

In *Pitcairnia* species, the total amino acid concentration in nectar ranged from 0.1 to 80 mM, with the highest concentrations found in species with reddish flowers or bracts, where mainly the alanine concentration was increased (Figure 3C and Figure 4A). Hummingbirds have been described as the main pollinators of these *Pitcairnia* species [55,60]. Hummingbirds appear to prefer lower concentrations of amino acids in nectar, because they can generally attain much more nitrogen from feeding on insects [17,61]. The high amino acid concentrations in the nectar of *Pitcairnia* species with reddish flower color, therefore, hint to pollination by more than only hummingbirds. Higher concentrations of amino acids in nectar have been found in plant species pollinated by the passerine birds, for example in species of the genera *Erythrina*, *Nicotiana*, or *Fritillaria* [5,14,24]. For several plant species, it has been described that flowers are visited by several functional pollinator groups, but with varying effectiveness [62,63,64]. *Pitcairnia angustifolia* flowers have been proven to be visited not only by hummingbirds but also by passerines such as *Coereba flaveola* [65]. *Coereba flaveola* (bananaquit) belongs to the tanager family (Thraupidae), which includes several nectar-feeding species. It accounts for 12% of the Neotropical avifauna [66]. Following hummingbirds, tanagers are generally the second most frequent visitors of bromeliads [67]. *Coereba flaveola* has been described as a predatory pollinator, that is a less effective pollinator of *P. angustifolia* than long-billed hummingbirds. However, when visitation rate was considered, there was no evidence of higher effectiveness of hummingbirds compared to the passerines [65]. Bananaquits have high nitrogen requirements, thus nectar which is low in amino acids limits their diet [68]. Therefore, it may be that other primarily trochilophilous *Pitcairnia* species with elevated amino acid concentrations in the nectar are visited by passerine birds as additional pollinators.

In addition to birds as pollinators for *Pitcairnia* species, butterflies are also possible pollinators, as the nectar of butterfly-pollinated flowers contain higher levels of amino acids than nectar of flowers pollinated by most other animal types [17]. The uptake of amino acids from floral nectar enhances reproduction of female and male butterflies and a co-evolutionary pollination process between butterflies and flowers was suggested [18,69,70]. So far, no evidence of pollination by butterflies has been found for *Pitcairnia* species [40], which, however, does not mean that this possibility must be ruled out.

An alternative explanation for the high amino acid concentrations in nectar of most hummingbird-pollinated *Pitcairnia* species with reddish flowers or bracts may be that these hummingbird species prefer higher amino acid concentrations in nectar than other hummingbird species. It is commonly discussed that one reason for the diversification of bromeliads may be the specialization for different hummingbird species [60]. Another explanation may be that the correlation between the amino acid concentration in nectar and the type of avian pollinator is not as strong as previously postulated [14].

The analyzed *Pitcairnia* species with yellow/white sepals and petals are likely to be pollinated by hummingbirds (Supplementary Table S1), and the nectar of these species contained only low concentrations of amino acids (Figure 3C). The reasons as to why nectar of hummingbird-pollinated species with reddish flowers and bracts contain higher amino acid concentrations than the nectar of species with yellow/white flowers are yet to be investigated. 

Night-flowering *Pitcairnia* species with greenish/white sepals and petals are pollinated by bats [55]. In general, bat-pollinated bromeliads produce high volumes of nectar and the nectar contains lower amino acid concentrations than the nectar of hummingbird-pollinated species [4]. This also applies to *Pitcairnia* species pollinated by bats (Figure 3C). In the nectar of night-flowering *Pitcairnia* species, glutamine was the main amino acid; its portion of the total amino acids in nectar was highest in *P. recurvata* as up to 50%. (Table 1). High concentrations of glutamine have also been found in the nectar of other night-flowering species pollinated by bats, for example *Nicotiana otophora* [5]. The reason for this could be that there is less carbon available for amino acid synthesis at night than during the light period, and glutamine (as well as the other amide asparagine) has a lower carbon to nitrogen ratio compared to most other amino acids [5].

With the results of the comparative analysis with BayesTraits, it could be shown that the presence of high amino acid concentrations in nectar (>10 mM) and the presence of reddish flower or bracts color are likely to have evolved together. For this method, groups had to be simplified to only two, namely “red” (reddish sepals and petals, reddish bracts) and “non-red” (yellow/white and greenish/white flowers), and there is no distinction within these groups. More detailed analyses of the data using PGLS regression considering the four color groups (reddish sepals and petals, reddish bracts, yellow/white sepals and petals, greenish/white sepals and petals) and actual amino acid concentrations showed that phylogeny does not have a direct influence on the variations of flower color and amino acid concentrations. Using detailed analysis (PGLS), the changes of the traits (color and amino acid concentration) are independent of the phylogeny, but there is still a relation between the two features “red” and “high amino acid concentration”. 

### 4.2. Do High Alanine Concentrations in Nectar Reflect Hypoxic Environments in Nectaries?

Alanine is involved in several metabolic pathways, and, in addition, oxygen deficiency caused by flooding has been shown to lead to an increase in alanine concentration in various plant tissues [71,72,73]. Plants’ reactions to hypoxia (low oxygen) include inhibition of oxidative phosphorylation as well as an increase in the glycolysis for ATP production and fermentation pathways for the required regeneration of NAD^+^ [74]. However, the conversion of pyruvate (the last product of the glycolysis) to alanine does not directly regenerate NAD^+^, and it remains unknown as to how the production of alanine supports anoxic metabolism [75]. 

High concentrations of alanine were also found in the nectars of *Cucurbita pepo*, which could be explained by possible local hypoxic environments in the nectaries [31]. In addition to flooding or other environmental conditions, the reason for hypoxia can also be due to obstruction of oxygen diffusion by the tissue structure [74]. In the *Pitcairnia* species analyzed, the septal nectaries are located near the base of the flowers, and the nectaries have dense cell packing [29]. Hence, it cannot be ruled out that there are local hypoxia environments in the septal nectaries which correlate with high alanine concentrations in nectar (Figure 4A). Alanine is dominant in the amino acid profile in the nectar of those *Pitcairnia* species with high total amino acid concentrations (Figure 4A, Table 1). However, flower length, as a measure of how deeply nectaries and nectar are buried in the flower, did not correlate with the amino acid (alanine) concentration in nectar (Figure 2C), and the oxygen conditions were probably similar in the nectaries of all *Pitcairnia* species. Therefore, hypoxia is unlikely to be the reason for high alanine concentrations in the nectar of species with reddish flowers or bracts and for low concentrations in species with yellow/white or greenish/white flowers (Figure 4A).

In addition to alanine, β-alanine, a non-proteinogenic amino acid and precursor of various biochemical molecules, also occurs in plants as a stress-response molecule, for example in heat or hypoxia [76]. The metabolism of L-alanine and β-alanine are linked because L-alanine can act as an amino donor in the synthesis of β-alanine by the β-alanine/L-alanine aminotransferase. Increased expression of this enzyme was found in *Arabidopsis* roots in response to hypoxia [76]. In leaves, nectaries, and nectar of the *Pitcairnia* species analyzed, the concentration of β-alanine was very low (leaves 0–1.1 mM; nectaries 0–1.2 mM; nectar 0–0.3 mM), and in about half of the species no β-alanine was detectable. Furthermore, no significant differences were found in the concentration of β-alanine in nectaries and nectar of the four flower groups (Figure 4E). Therefore, it can be assumed that β-alanine plays neither a special role in the interaction between plants and pollinators nor as a hypoxia-response molecule in *Pitcairnia* species.

### 4.3. Origin of the Amino Acids Contained in Nectar, Especially Alanine and Glutamine

Nectar compounds are produced and secreted by nectaries [77]. Several models have been developed showing the nectar production by nectaries, with a main focus on sugars, while there are very few studies focusing on amino acid production [2,31]. All proteinogenic amino acids were found in the nectar of the *Pitcairnia* species, but alanine was the most abundant, followed by glutamine (Figure 4, Table 1). In general, the two mentioned amino acids together with glutamate, aspartate, asparagine, and serine are the main amino acids in the nectar of several plant species [4,14,24]. These non-essential amino acids generally play a central role in nitrogen metabolism in various plant tissues, and they are the main amino acids in the phloem sap of most plant species [43].

Compared to the phloem sap, the total amino acid concentration in nectar is one or two orders of magnitude lower, suggesting that the amino acids are probably retained in the nectaries during nectar secretion [34,78]. This becomes also visible when comparing the total amino acid concentrations in nectaries and nectar of the *Pitcairnia* species analyzed, as the concentration is much higher in nectaries than in nectar (Figure 3C). The difference between nectaries and nectar depends on the flower color and/or pollinator type: the amino acid concentration in nectaries of *Pitcairnia* species with reddish flowers or bracts was about 4 to 5 times higher than in nectar, in species with yellow/white flowers about 25 times higher, and in species with greenish/white flowers about 55 times higher (Figure 3C). The same applies to individual amino acids, the concentration of which was also always higher in nectaries than in nectar, with the exception of alanine (Figure 4A). The alanine concentration in the nectar of *Pitcairnia* species with reddish flowers or bracts was similar to that in nectaries (Figure 4A), and, furthermore, higher alanine concentrations in nectar correlated with higher concentrations in nectaries (correlation factor = 0.62, *p* ≤ 0.001). Therefore, active regulatory mechanisms are required in nectaries that retain most of the amino acids and allow for selective secretion of specific amino acids into the nectar.

In addition to the presence of amino acids in nectar due to their transport in the phloem sap to the nectaries, the amino acids can also be synthesized in the nectaries themselves [31]. This could be the case particularly for alanine, since a four times higher activity of AlaAT was found in nectaries than in the leaves of the analyzed *Pitcairnia* species (Figure 5E,G). An increase in AlaAT activity was also found in *Cucurbita pepo* nectaries during nectar secretion [31]. Despite the higher alanine concentrations in the nectar in *Pitcairnia* species with reddish flowers or bracts compared to the nectar of species with yellow/white or greenish/white flowers, the detected activity of AlaAT in leaves or nectaries was similar in both groups (Figure 5E–H). It was also shown in *Cucurbita pepo* that the measured activity of AlaAT was not correlated with the alanine concentration in nectaries because a three-fold reduction of the AlaAT activity only led to a slight reduction in the alanine content in nectaries [31]. The reason for this could be that the maximum AlaAT activity in the tissue was detected through employing the enzyme assay, but that in vivo the activity of the enzyme is regulated in the nectaries [71]. Furthermore, different AlaAT isoforms, which are involved in different cellular processes, have been localized in different subcellular compartments [79,80]. The enzyme assay used in this study may have detected the activities of all isoforms in the nectaries, regardless of whether they are involved in nectar production or not. 

The activity of GS was much higher in the leaves than in nectaries of the analyzed *Pitcairnia* species (Figure 5A,C). Therefore, it can be assumed that glutamine is produced in rather small amounts in nectaries and mainly in the other parts of the plant and transported to the nectaries. This assumption is supported by the fact that glutamine is one of the most abundant amino acids in the phloem sap of various plant species [81]. Glutamine also plays an indirect role in alanine metabolism in nectaries, as glutamine and 2-oxoglutarate can be converted to glutamate, which can be used by AlaAT as an amino group donor in alanine synthesis [82,83]. However, glutamine concentration in nectaries was similar among species of all flower-color groups (Figure 4B), and no correlation was found between alanine and glutamine concentrations.

Pyruvate and malate are direct or indirect carbon skeletons for the synthesis of amino acid. Pyruvate is the end product of the glycolysis or it can be produced from malate by the NAD-dependent malic enzyme [84,85]. Pyruvate, along with glutamate, can be reversibly converted to alanine and 2-oxoglutarate catalyzed by AlaAT [86]. This corresponds to the lower pyruvate concentrations in the nectaries of those species with higher alanine concentrations, species with reddish flowers or bracts, and species with yellow/white sepals and petals, and higher pyruvate concentrations in the nectaries of those species with lower alanine concentrations, such as species with greenish/white sepals and petals (Figure 4A and Figure 6A). 

Very little is known so far about the transport of amino acids from the nectaries into the nectar [2]. Amino acid transporters that may be involved in nectar secretion, for example, have not yet been clearly identified [87].

## 5. Conclusions

In summary, the study of *Pitcairnia* species has provided new insights into the function and origin of amino acids in nectar. A large amount of the amino acids in nectar is probably produced in the plant and transported via the phloem to the nectaries and are subsequently secreted into the nectar. To provide the floral nectar with sufficient amino acids, some amino acids must additionally be produced de novo in the nectaries. This may be true for the non-essential amino acid alanine in *Pitcairnia* species. Nevertheless, nitrogen metabolism in the nectaries is still not fully elucidated. The possible involvement of transporters in the secretion of nectar is also uncertain.

Alanine is the most abundant amino acid in *Pitcairnia* species. The hypothesis that alanine is responsible for countering hypoxia in the nectaries can be rejected for these species. It is more likely that *Pitcairnia* species with reddish flowers have increased concentrations of alanine and also glutamine in the nectar, so that they are pollinated by passerine birds, butterflies, or further pollinators in addition to hummingbirds.

## Figures and Tables

**Table 1:** Percentage of different amino acids in nectar of *Pitcairnia* species. In addition to individual amino acids (Ala = alanine; Gln = glutamine; Glu = glutamic acid; Asn = asparagine; Asp = aspartic acid; Ser = serine; Pro = proline), the following groups were listed: essential amino acids (arginine, histidine, isoleucine, leucine, lysine, methionine, phenylalanine, threonine, tryptophan, valine), residual amino acids (glycine, tyrosine), non-proteinogenic amino acids (β-alanine, γ-aminobutyric acid, taurine).

	Ala [%]	Gln [%]	Glu [%]	Asn [%]	Asp [%]	Ser [%]	Pro [%]	Essential Amino Acids [%]	Residual Amino Acids [%]	Non-Proteinogenic Amino Acids [%]
Reddish sepals and petals										
*P. angustifolia*	42.6 ± 0.8	13.7 ± 0.2	0.5 ± 0.0	8.5 ± 0.2	0.2 ± 0.0	12.3 ± 0.2	0.2 ± 0.1	12.3 ± 1.2	9.7 ± 0.4	0.0 ± 0.0
*P. bromeliifolia*	64.9 ± 1.3	10.3 ± 1.7	0.3 ± 0.3	2.1 ± 0.1	0.2 ± 0.2	9.7 ± 0.1	0.7 ± 0.1	8.3 ± 0.9	3.4 ± 0.3	0.0 ± 0.0
*P. carnososepala*	84.1 ± 1.3	0.6 ± 0.1	0.0 ± 0.0	0.2 ± 0.0	0.0 ± 0.0	8.7 ± 0.8	0.1 ± 0.0	3.6 ± 0.3	2.3 ± 0.2	0.3 ± 0.1
*P. chiriquensis*	26.5 ± 3.5	17.5 ± 2.4	2.8 ± 0.2	3.6 ± 1.7	3.4 ± 0.0	8.8 ± 1.4	0.5 ± 0.9	21.1 ± 7.0	2.5 ± 0.5	13.3 ± 4.4
*P. corallina*	70.6 ± 3.4	8.2 ± 2.1	0.0 ± 0.0	1.1 ± 0.3	0.1 ± 0.1	10.7 ± 1.0	0.1 ± 0.0	4.6 ± 1.7	4.4 ± 1.1	0.2 ± 0.0
*P. echinata var. vallensis*	55.1 ± 5.0	9.1 ± 2.8	0.2 ± 0.1	8.4 ± 6.8	0.2 ± 0.2	15.1 ± 1.8	0.2 ± 0.1	9.7 ± 4.1	1.8 ± 0.7	0.1 ± 0.1
*P. integrifolia*	30.9 ± 25.5	19.7 ± 8.7	2.7 ± 3.0	23.2 ± 21.1	1.6 ± 0.8	7.0 ± 5.3	1.7 ± 3.0	10.2 ± 5.6	2.3 ± 1.5	0.8 ± 1.3
*P. jimenezii*	41.0 ± 3.2	33.8 ± 1.6	0.3 ± 0.0	6.7 ± 5.3	0.3 ± 0.2	8.9 ± 0.5	0.4 ± 0.2	6.2 ± 0.4	2.2 ± 0.1	0.2 ± 0.0
*P. nigra var. nigra*	76.9 ± 0.1	2.0 ± 0.0	0.2 ± 0.0	7.2 ± 0.2	0.1 ± 0.1	5.7 ± 0.2	0.0 ± 0.0	2.8 ± 0.1	5.0 ± 0.1	0.0 ± 0.0
*P. orchidifolia*	46.1 ± 8.7	17.7 ± 2.4	1.0 ± 0.6	3.5 ± 2.7	1.1 ± 1.0	17.1 ± 2.0	0.1 ± 0.0	8.3 ± 3.5	5.1 ± 2.0	0.0 ± 0.0
*P. poeppigiana*	72.1 ± 2.1	6.0 ± 0.6	0.6 ± 0.2	3.9 ± 0.6	0.4 ± 0.2	7.8 ± 0.0	1.1 ± 0.5	4.5 ± 0.6	3.6 ± 0.1	0.0 ± 0.0
*P. rubronigriflora*	29.9 ± 10.6	29.8 ± 8.7	0.7 ± 0.5	16.3 ± 8.8	1.5 ± 0.2	8.1 ± 1.9	0.5 ± 0.4	8.4 ± 4.4	2.6 ± 1.2	2.4 ± 1.2
*P. spicata*	35.5 ± 2.0	23.9 ± 1.7	0.5 ± 0.0	20.1 ± 1.3	0.5 ± 0.0	9.2 ± 0.3	1.3 ± 0.9	6.7 ± 1.1	1.8 ± 0.2	0.6 ± 0.5
*P. sprucei*	59.9 ± 28.6	10.5 ± 14.4	0.6 ± 0.6	9.2 ± 10.3	0.8 ± 1.1	9.0 ± 29	0.6 ± 0.2	7.3 ± 6.6	2.2 ± 1.1	0.0 ± 0.0
*P. utcubambensis*	59.4 ± 4.4	10.2 ± 0.8	0.2 ± 0.1	3.8 ± 1.3	0.2 ± 0.0	10.9 ± 1.2	0.4 ± 0.2	11.6 ± 5.0	3.1 ± 0.8	0.2 ± 0.3
Mean value:	53.0 ± 8.5	14.2 ± 9.6	0.7 ± 0.9	7.8 ± 6.9	0.7 ± 0.9	9.9 ± 3.0	0.5 ± 0.5	8.4 ± 4.5	3.5 ± 2.0	1.2 ± 3.4
Reddish bracts										
*P. atrorubens*	56.9 ± 10.1	9.3 ± 2.2	0.8 ± 0.5	9.0 ± 9.5	0.5 ± 0.4	13.1 ± 1.7	0.0 ± 0.0	7.5 ± 3.0	2.3 ± 0.5	0.6 ± 0.2
*P. flagellaris*	73.4 ± 2.7	3.7 ± 0.5	0.1 ± 0.1	3.8 ± 2.6	0.0 ± 0.0	8.0 ± 0.5	0.3 ± 0.0	6.2 ± 0.4	4.0 ± 0.4	0.4 ± 0.1
*P. imbricata*	56.6 ± 8.9	12.6 ± 3.1	0.4 ± 0.3	6.6 ± 2.3	0.3 ± 0.2	9.5 ± 3.3	0.9 ± 0.2	7.7 ± 6.3	3.9 ± 0.6	1.6 ± 0.6
*P. maidifolia*	23.6 ± 5.9	26.2 ± 3.2	0.7 ± 0.1	24.3 ± 9.8	0.4 ± 0.1	12.6 ± 1.0	0.1 ± 0.1	9.7 ± 1.2	1.8 ± 0.3	0.5 ± 0.1
*P. oliva-estevae*	1.3 ± 0.2	7.2 ± 1.2	1.1 ± 0.2	67.1 ± 3.6	1.2 ± 0.3	2.0 ± 0.2	2.0 ± 0.4	14.5 ± 6.2	1.2 ± 0.1	2.5 ± 0.5
*P. wendlandii*	72.0 ± 1.6	2.8 ± 0.5	0.7 ± 0.3	2.6 ± 0.1	1.2 ± 0.5	10.6 ± 0.2	2.8 ± 0.8	5.2 ± 1.5	2.0 ± 0.3	0.0 ± 0.0
Mean value:	47.3 ± 28.8	10.3 ± 8.6	0.6 ± 0.3	18.9 ± 24.9	0.6 ± 0.5	9.3 ± 4.1	1.0 ± 1.1	8.5 ± 3.3	2.5 ± 1.2	0.9 ± 0.9
Yellow/white sepals and petals										
*P. albolutea*	6.3 ± 4.6	6.9 ± 1.8	4.2 ± 2.3	28.6 ± 6.4	2.7 ± 0.8	5.2 ± 1.5	1.5 ± 0.8	34.9 ± 9.9	9.8 ± 2.1	0.0 ± 0.0
*P. altensteinii var. altensteinii*	1.9 ± 0.8	10.1 ± 5.6	2.9 ± 0.3	12.9 ± 7.0	2.1 ± 0.6	3.7 ± 0.5	9.0 ± 5.6	49.7 ± 16.2	2.9 ± 1.1	4.8 ± 3.6
*P. breedlovei*	8.5 ± 2.1	52.3 ± 7.1	3.8 ± 2.5	6.1 ± 3.6	3.1 ± 2.1	5.8 ± 1.5	1.6 ± 0.4	9.7 ± 4.6	2.2 ± 1.0	6.9 ± 3.8
*P. capixaba*	1.4 ± 0.8	7.5 ± 3.0	4.7 ± 0.9	2.6 ± 1.3	4.0 ± 1.1	3.8 ± 0.0	2.0 ± 1.4	49.8 ± 17.7	18.4 ± 5.8	6.0 ± 1.2
*P. chiapensis*	43.0 ± 1.7	12.5 ± 0.6	0.8 ± 1.5	0.4 ± 0.1	0.0 ± 0.0	17.9 ± 0.2	0.2 ± 0.2	21.5 ± 5.5	3.7 ± 0.7	0.0 ± 0.0
*P. sceptrigera*	53.9 ± 1.2	11.6 ± 0.3	1.5 ± 0.6	5.6 ± 0.4	1.0 ± 0.1	10.4 ± 0.7	1.2 ± 0.3	10.8 ± 0.6	3.1 ± 0.3	0.8 ± 0.2
*P. suaveolens*	8.0 ± 0.7	22.6 ± 3.3	8.4 ± 6.1	10.1 ± 5.5	5.1 ± 2.0	7.7 ± 1.3	1.1 ± 1.2	25.5 ± 15.0	4.2 ± 2.4	7.4 ± 2.4
Mean value:	17.6 ± 21.5	17.6 ± 16.2	3.8 ± 2.5	9.5 ± 9.4	2.6 ± 1.7	7.8 ± 5.0	2.3 ± 3.0	28.8 ± 16.7	6.3 ± 5.9	3.7 ± 3.3
Greenish/white sepals and petals										
*P. longissimiflora*	1.7 ± 0.3	16.4 ± 12.1	21.9 ± 8.5	21.9 ± 20.1	9.9 ± 4.5	6.1 ± 2.2	14.3 ± 15.7	7.6 ± 3.4	1.5 ± 1.3	4.0 ± 3.5
*P. recurvata*	8.1 ± 7.1	46.4 ± 29.4	9.9 ± 1.9	9.9 ± 17.2	8.4 ± 8.3	2.9 ± 1.8	6.6 ± 5.0	4.7 ± 4.4	0.0 ± 0.0	1.7 ± 2.3
Mean value:	4.9 ± 4.5	31.4 ± 21.2	15.9 ± 8.5	15.9 ± 8.5	9.2 ± 1.1	4.5 ±2.3	10.4 ± 5.5	6.2 ± 2.0	0.7 ± 1.0	2.9 ± 1.7

## Data Availability

Data are contained within the article and supplementary materials.

## Supplementary Materials

The following supporting information can be downloaded at https://www.mdpi.com/article/10.3390/plants13010023/s1, Table S1: Main features of all examined *Pitcairnia* species; Table S2: Results of the PERMANOVA and PERMDISP of three component groups: (A) amino acids, (B) sugars and inorganic ions, (C) Alanine. Data on leaves, nectaries, and nectar of four *Pitcairnia* species with red flowers or bracts and four *Pitcairnia* species with yellow/white or greenish/white flowers were used for the calculations. The significance level (*) for both methods is *p* ≤ 0.001; Table S3: Ratio of different compounds in leaves, nectaries, and nectar of *Pitcairnia* species separated by flower color; Figure S1: The inflorescence of the *Pitcairnia* species were separated into three types. *Pitcairnia grafii* corresponds to raceme inflorescence (A) *Pitcairnia flagellaris* corresponds to spike inflorescence (B) *Pitcairnia echinata var. vallensis* corresponds to panicle inflorescence (C). In each case the bracts, sepals and petals are labeled in the pictures. In addition to the picture of each inflorescence, a schematic illustration of the inflorescence is also provided; Figure S2: Comparison of total amino acid concentration in nectar of two *Pitcairnia* species from different botanical gardens. *Pitcairnia imbricata* was compared using the sites in Berlin and Stuttgart, and *Pitcairnia sceptrigera* was compared using the sites in Berlin and Heidelberg. Different letters represent significant differences in total amino acid concentration, respectively, between the botanical gardens (Tukey’s HSD; *p* < 0.05).

## References

[1] Kessler D., Bhattacharya S., Diezel C., Rothe E., Gase K., Schöttner M., Baldwin I.T. (2012). Unpredictability of nectar nicotine promotes outcrossing by hummingbirds in *Nicotiana attenuata*. Plant J..

[2] Nicolson S.W. (2022). Sweet solutions: Nectar chemistry and quality. Philos. Trans. R. Soc. Lond. Ser. B Biol. Sci..

[3] Proctor M., Yeo P., Lack A. (1996). The Natural History of Pollination.

[4] Göttlinger T., Schwerdtfeger M., Tiedge K., Lohaus G. (2019). What do nectarivorous bats like? Nectar composition in Bromeliaceae with special emphasis on bat-pollinated species. Front. Plant Sci..

[5] Tiedge K., Lohaus G. (2017). Nectar sugars and amino acids in day- and night-flowering *Nicotiana* species are more strongly shaped by pollinators’ preferences than organic acids and inorganic ions. PLoS ONE.

[6] Witt T., Jürgens A., Gottsberger G. (2013). Nectar sugar composition of european Caryophylloideae (Caryophyllaceae) in relation to flower length, pollination biology and phylogeny. J. Evol. Biol..

[7] Göttlinger T., Lohaus G. (2020). Influence of light, dark, temperature and drought on metabolite and ion composition in nectar and nectaries of an epiphytic bromeliad species (*Aechmea fasciata*). Plant Biol..

[8] Waser N.M., Price M.V. (2016). Drought, pollen and nectar availability, and pollination success. Ecology.

[9] Baker H.G., Baker I. (1973). Amino acids in nectar and their evolutionary significance. Nature.

[10] Heil M. (2011). Nectar: Generation, regulation and ecological functions. Trends Plant Sci..

[11] Lohaus G., Schwerdtfeger M. (2014). Comparison of sugars, iridoid glycosides and amino acids in nectar and phloem sap of *Maurandya barclayana*, *Lophospermum erubescens*, and *Brassica napus*. PLoS ONE.

[12] Gardener M.C., Gillman M.P. (2001). Analyzing variability in nectar amino acids: Composition is less variable than concentration. J. Chem. Ecol..

[13] Nicolson S.W. (2007). Amino acid concentrations in the nectars of Southern African bird-pollinated flowers, especially aloe and erythrina. J. Chem. Ecol..

[14] Petanidou T., van Laere A., Ellis W.N., Smets E. (2006). What shapes amino acid and sugar composition in Mediterranean floral nectars?. Oikos.

[15] Gijbels P., Ceulemans T., van den Ende W., Honnay O. (2015). Experimental fertilization increases amino acid content in floral nectar, fruit set and degree of selfing in the orchid *Gymnadenia conopsea*. Oecologia.

[16] Nepi M. (2014). Beyond nectar sweetness: The hidden ecological role of non-protein amino acids in nectar. J. Ecol..

[17] Baker H.G., Baker I. (1986). The occurrence and significance of amino acids in floral nectar. Plant Syst. Evol..

[18] Mevi-Schutz J., Erhardt A. (2005). Amino acids in nectar enhance butterfly fecundity: A long-awaited link. Am. Nat..

[19] Inouye D.W., Waller G.D. (1984). Responses of honey bees (*Apis mellifera*) to amino acid solutions mimicking floral nectars. Ecology.

[20] Carter C., Shafir S., Yehonatan L., Palmer R.G., Thornburg R. (2006). A novel role for proline in plant floral nectars. Naturwissenschaften.

[21] Waller G.D. (1972). Evaluating responses of honey bees to sugar solutions using an artificial-flower feeder. Ann. Entomol. Soc. Am..

[22] Teulier L., Weber J.M., Crevier J., Darveau C.A. (2016). Proline as a fuel for insect flight: Enhancing carbohydrate oxidation in hymenopterans. Proc. R. Soc. B.

[23] Rodríguez-Peña N., Stoner K.E., Ayala-Berdon J., Flores-Ortiz C.M., Duran A., Schondube J.E. (2013). Nitrogen and amino acids in nectar modify food selection of nectarivorous bats. J. Anim. Ecol..

[24] Roguz K., Bajguz A., Chmur M., Gołębiewska A., Roguz A., Zych M. (2019). Diversity of nectar amino acids in the *Fritillaria* (Liliaceae) genus: Ecological and evolutionary implications. Sci. Rep..

[25] Vandelook F., Janssens S.B., Gijbels P., Fischer E., van den Ende W., Honnay O., Abrahamczyk S. (2019). Nectar traits differ between pollination syndromes in Balsaminaceae. Ann. Bot..

[26] Lin I.W., Sosso D., Chen L.Q., Gase K., Kim S.G., Kessler D., Klinkenberg P.M., Gorder M.K., Hou B.H., Qu X.Q. (2014). Nectar secretion requires sucrose phosphate synthases and the sugar transporter SWEET9. Nature.

[27] Peng Y.B., Li Y.Q., Hao Y.J., Xu Z.H., Bai S.N. (2004). Nectar production and transportation in the nectaries of the female *Cucumis sativus* L. flower during anthesis. Protoplasma.

[28] Ren G., Healy R.A., Klyne A.M., Horner H.T., James M.G., Thornburg R.W. (2007). Transient starch metabolism in ornamental tobacco floral nectaries regulates nectar composition and release. Plant Sci..

[29] Stahl J.M., Nepi M., Galetto L., Guimarães E., Machado S.R. (2012). Functional aspects of floral nectar secretion of *Ananas ananassoides*, an ornithophilous bromeliad from the Brazilian savanna. Ann. Bot..

[30] Mosti S., Ross Friedman C., Pacini E., Brighigna L., Papini A. (2013). Nectary ultrastructure and secretory modes in three species of *Tillandsia* (Bromeliaceae) that have different pollinators. Botany.

[31] Solhaug E.M., Roy R., Venterea R.T., Carter C.J. (2021). The role of alanine synthesis and nitrate-induced nitric oxide production during hypoxia stress in *Cucurbita pepo* nectaries. Plant J..

[32] Elliott W. (1953). Isolation of glutamine synthetase and glutamotransferase from green peas. J. Biol. Chem..

[33] Rech J., Crouzet J. (1974). Partial purification and initial studies of the tomato L-alanine:2-oxoglutarate aminotransferase. Biochim. Biophys. Acta.

[34] Göttlinger T., Lohaus G. (2022). Comparative analyses of the metabolite and ion concentrations in nectar, nectaries, and leaves of 36 bromeliads with different photosynthesis and pollinator types. Front. Plant Sci..

[35] Givnish T.J., Millam K.C., Evans T.M., Hall J.C., Chris Pires J., Berry P.E., Sytsma K.J. (2004). Ancient vicariance or recent long-distance dispersal? Inferences about phylogeny and South American–African disjunctions in Rapateaceae and Bromeliaceae Based on ndhF sequence data. Int. J. Plant Sci..

[36] Givnish T.J. (2007). Phylogeny, adaptive radiation and historical biogeography of Bromeliaceae inferred from ndhF sequence data. Aliso.

[37] Crayn D.M., Winter K., Schulte K., Smith J.A.C. (2015). Photosynthetic pathways in Bromeliaceae: Phylogenetic and ecological significance of CAM and C 3 based on carbon isotope ratios for 1893 species. Bot. J. Linn. Soc..

[38] Aguilar-Rodríguez P.A., Tschapka M., García-Franco J.G., Krömer T., MacSwiney González M.C. (2019). Bromeliads going batty: Pollinator partitioning among sympatric chiropterophilous Bromeliaceae. AoB Plants.

[39] Benzing D.H. (2000). Bromeliaceae: Profile of an Adaptive Radiation.

[40] Krömer T., Kessler M., Herzog S.K. (2006). Distribution and flowering ecology of bromeliads along two climatically contrasting elevational transects in the Bolivian Andes. Biotropica.

[41] Saraiva D.P., Mantovani A., Forzza R.C. (2015). Insights into the Evolution of *Pitcairnia* (Pitcairnioideae-Bromeliaceae), based on Morphological Evidence. Syst. Bot..

[42] Sajo M.G., Rudall P.J., Prychid C.J. (2004). Floral anatomy of Bromeliaceae, with particular reference to the evolution of epigyny and septal nectaries in commelinid monocots. Plant Syst. Evol..

[43] Lohaus G. (2022). Review primary and secondary metabolites in phloem sap collected with aphid stylectomy. J. Plant Physiol..

[44] de La Barrera E., Nobel P.S. (2004). Nectar: Properties, floral aspects, and speculations on origin. Trends Plant Sci..

[45] Tiedge K., Lohaus G. (2018). Nectar sugar modulation and cell wall invertases in the nectaries of day- and night-flowering *Nicotiana*. Front. Plant Sci..

[46] Riens B., Lohaus G., Winter H., Heldt H. (1994). Production and diurnal utilization of assimilates in leaves of spinach (*Spinacia oleracea* L.) and barley (*Hordeum vulgare* L.). Planta.

[47] Shapiro B.M., Stadtman E.R. (1970). Glutamine synthetase (*Escherichia coli*). Methods Enzymol..

[48] Schubert K. (2017). Systematik und Evolution der Gattung Pitcairnia L’Heritier (Bromeliaceae).

[49] Pagel M. (1994). Detecting correlated evolution on phylogenies: A general method for the comparative analysis of discrete characters. Proc. R. Soc. Lond. B Biol. Sci..

[50] Orme D., Freckleton R., Thomas G., Petzoldt T., Fritz S., Isaac N., Pearse W. (2013). Caper: Comparative Analyses of Phylogenetics and Evolution in R. R Package Version 1.0.1. https://CRAN.R-project.org/package=caper.

[51] Wei T., Simko V. R Package ’Corrplot’: Visualization of a Correlation Matrix 2021. https://github.com/taiyun/corrplot.

[52] Anderson M.J., Balakrishnan N., Colton T., Everitt B., Piegorsch W., Ruggeri F., Teugels J.L. (2014). Permutational Multivariate Analysis of Variance (PERMANOVA). Wiley StatsRef: Statistics Reference Online.

[53] Fenster C.B., Armbruster W.S., Wilson P., Dudash M.R., Thomson J.D. (2004). Pollination syndromes and floral specialization. Annu. Rev. Ecol. Evol. Syst..

[54] Abrahamczyk S., Kessler M., Hanley D., Karger D.N., Müller M.P.J., Knauer A.C., Keller F., Schwerdtfeger M., Humphreys A.M. (2017). Pollinator adaptation and the evolution of floral nectar sugar composition. J. Evol. Biol..

[55] Krömer T., Kessler M., Lohaus G., Schmidt-Lebuhn A.N. (2008). Nectar sugar composition and concentration in relation to pollination syndromes in Bromeliaceae. Plant Biol..

[56] Perret M. (2001). Nectar sugar composition in relation to pollination syndromes in Sinningieae (Gesneriaceae). Ann. Bot..

[57] Brzosko E., Bajguz A. (2019). Nectar composition in moth-pollinated *Platanthera bifolia* and *P. chlorantha* and its importance for reproductive success. Planta.

[58] Gardener M.C., Gillman M.P. (2002). The taste of nectar—A neglected area of pollination ecology. Oikos.

[59] Quintana-Rodríguez E., Ramírez-Rodríguez A.G., Ramírez-Chávez E., Molina-Torres J., Camacho-Coronel X., Esparza-Claudio J., Heil M., Orona-Tamayo D. (2018). Biochemical traits in the flower lifetime of a Mexican mistletoe parasitizing mesquite biomass. Front. Plant Sci..

[60] Kessler M., Abrahamczyk S., Krömer T. (2020). The role of hummingbirds in the evolution and diversification of Bromeliaceae: Unsupported claims and untested hypotheses. Bot. J. Linn. Soc..

[61] Hainsworth F.R., Wolf L.L. (1972). Crop volume, nectar concentration and hummingbird energetics. Comp. Biochem. Physiol. Part A Physiol..

[62] Rosas-Guerrero V., Quesada M., Armbruster W.S., Pérez-Barrales R., Smith S.D. (2011). Influence of pollination specialization and breeding system on floral integration and phenotypic variation in Ipomoea. Evolution.

[63] Waser N.M., Chittka L., Price M.V., Williams N.M., Ollerton J. (1996). Generalization in pollination systems, and why it matters. Ecology.

[64] Wendt T., Canela M.B., Gelli de Faria A.P., Rios R.I. (2001). Reproductive biology and natural hybridization between two endemic species of *Pitcairnia* (Bromeliaceae). Am. J. Bot..

[65] Fumero-Cabán J.J., Meléndez-Ackerman E.J. (2007). Relative pollination effectiveness of floral visitors of *Pitcairnia angustifolia* (Bromeliaceae). Am. J. Bot..

[66] Burns K.J., Shultz A.J., Title P.O., Mason N.A., Barker F.K., Klicka J., Lanyon S.M., Lovette I.J. (2014). Phylogenetics and diversification of tanagers (Passeriformes: Thraupidae), the largest radiation of Neotropical songbirds. Mol. Phylogenet. Evol..

[67] Rocha J. (2023). Neotropical bromeliads as food sources for birds: A systematic review and perspectives on the management of ecological interactions. Ibis.

[68] Michel P., Pérez-Emán J., Mata A. (2013). The Bananaquit, a neotropical passerine nectar feeding bird, has a high protein requirement relative to other nectarivorous birds. J. Ornithol..

[69] Cahenzli F., Erhardt A. (2012). Enhancing offspring quality or quantity? Different ways for using nectar amino acids in female butterflies. Oecologia.

[70] Cahenzli F., Erhardt A. (2013). Nectar amino acids enhance reproduction in male butterflies. Oecologia.

[71] Miyashita Y., Dolferus R., Ismond K.P., Good A.G. (2007). Alanine aminotransferase catalyses the breakdown of alanine after hypoxia in *Arabidopsis thaliana*. Plant J..

[72] Mustroph A., Barding G.A., Kaiser K.A., Larive C.K., Bailey-Serres J. (2014). Characterization of distinct root and shoot responses to low-oxygen stress in *Arabidopsis* with a focus on primary C- and N-metabolism. Plant Cell Environ..

[73] Streeter J.G., Thompson J.F. (1972). Anaerobic accumulation of gamma-aminobutyric acid and alanine in radish leaves (*Raphanus sativus*, L.). Plant Physiol..

[74] Diab H., Limami A.M. (2016). Reconfiguration of N metabolism upon hypoxia stress and recovery: Roles of alanine aminotransferase (AlaAT) and glutamate dehydrogenase (GDH). Plants.

[75] de Sousa C.A.F., Sodek L. (2002). The metabolic response of plants to oxygen deficiency. Braz. J. Plant Physiol..

[76] Parthasarathy A., Adams L.E., Savka F.C., Hudson A.O. (2019). The *Arabidopsis thaliana* gene annotated by the locus tag At3g08860 encodes alanine aminotransferase. Plant Direct.

[77] Roy R., Schmitt A.J., Thomas J.B., Carter C.J. (2017). Review: Nectar biology: From molecules to ecosystems. Plant Sci..

[78] Bertazzini M., Forlani G. (2016). Intraspecific variability of floral nectar volume and composition in rapeseed (*Brassica napus* L. var. *oleifera*). Front. Plant Sci..

[79] Duff S.M.G., Rydel T.J., McClerren A.L., Zhang W., Li J.Y., Sturman E.J., Halls C., Chen S., Zeng J., Peng J. (2012). The enzymology of alanine aminotransferase (AlaAT) isoforms from *Hordeum vulgare* and other organisms, and the HvAlaAT crystal structure. Arch. Biochem. Biophys..

[80] McAllister C.H., Facette M., Holt A., Good A.G. (2013). Analysis of the enzymatic properties of a broad family of alanine aminotransferases. PLoS ONE.

[81] Lohaus G., Moellers C. (2000). Phloem transport of amino acids in two *Brassica napus* L. genotypes and one *B. carinata* genotype in relation to their seed protein content. Planta.

[82] Chatt E.C., Mahalim S.N., Mohd-Fadzil N.A., Roy R., Klinkenberg P.M., Horner H.T., Hampton M., Carter C.J., Nikolau B.J. (2021). Nectar biosynthesis is conserved among floral and extrafloral nectaries. Plant Physiol.

[83] Forde B.G., Lea P.J. (2007). Glutamate in plants: Metabolism, regulation, and signalling. J. Exp. Bot..

[84] Pratelli R., Pilot G. (2014). Regulation of amino acid metabolic enzymes and transporters in plants. J. Exp. Bot..

[85] Sweetlove L.J., Beard K.F.M., Nunes-Nesi A., Fernie A.R., Ratcliffe R.G. (2010). Not just a circle: Flux modes in the plant TCA cycle. Trends Plant Sci..

[86] Rocha M., Sodek L., Licausi F., Hameed M.W., Dornelas M.C., van Dongen J.T. (2010). Analysis of alanine aminotransferase in various organs of soybean (*Glycine max*) and in dependence of different nitrogen fertilisers during hypoxic stress. Amino Acids.

[87] Borghi M., Fernie A.R. (2017). Floral metabolism of sugars and amino acids: Implications for pollinators’ preferences and seed and fruit set. Plant Physiol..

