# Supplementary material for: Origin and Function of Amino Acids in Nectar and Nectaries of Pitcairnia Species with Particular Emphasis on Alanine and Glutamine
Source: Plants (Basel). 2023 Dec 20;13(1):23. doi: 10.3390/plants13010023 (PMC10780904; doi:10.3390/plants13010023)
Supplement: Supplementary file 1 [file plants-13-00023-s001.zip › Supplementary Table S1-S3.pdf]

Supplementary Material

Origin and function of amino acids in nectar and nectaries of *Pitcairnia* species with particular emphasis on alanine and glutamine

Author: Thomas Göttlinger*, Gertrud Lohaus

***Correspondence:** Thomas Göttlinger (goettlinger@uni-wuppertal.de)

Supplementary Table S1: Main features of all examined *Pitcairnia* species.

Species	Botanical garden	Main pollinator	Inflorescence type	Flower length [cm]	Floral bracts	Flower sepal	Flower petal	Group	Leaf samples	Nectary samples	Nectar samples	Enzyme assay
P. albolutea	Berlin	tro	spike	8.0	greenish	yellow	white	yellow/white sepals& petals	✓	✓	✓	-
P. altensteinii var. altensteinii	Berlin	tro	spike	5.5	yellow	yellow	white	yellow/white sepals& petals	✓	-	✓	-
P. angustifolia	Berlin	tro	raceme	5.5	greenish	reddish	reddish	reddish sepals & petals	✓	✓	✓	✓
P. atrorubens	Heidelberg	tro	spike	4.0	reddish	yellow	yellow	reddish bracts	✓	-	✓	-
P. breedlovei	Heidelberg	tro	raceme	5.0	reddish	yellow	yellow	yellow/white sepals& petals	✓	-	✓	-
P. bromeliifolia	Berlin	tro	raceme	4.5	reddish	reddish	reddish	reddish sepals & petals	✓	-	✓	-
P. capixaba	Berlin	tro	raceme	6.5	greenish	yellow	white	yellow/white sepals& petals	✓	✓	✓	✓
P. carnososepala	Berlin	tro	raceme	7.5	reddish	reddish	reddish	reddish sepals & petals	✓	-	✓	-
P. chiapensis	Berlin	tro	raceme	5.0	greenish	yellow	yellow	yellow/white sepals& petals	✓	-	✓	-
P. chiriquensis	Berlin	tro	panicle	4.5	reddish	reddish	reddish	reddish sepals & petals	✓	-	✓	-
P. corallina	Berlin	tro	raceme	7.0	reddish	reddish	reddish	reddish sepals & petals	✓	-	✓	-
P. echinata var. vallensis	Berlin	tro	panicle	4.5	greenish	reddish	reddish	reddish sepals & petals	✓	-	✓	-
P. flagellaris	Berlin	tro	spike	6.5	reddish	yellow	yellow	reddish bracts	✓	-	✓	-
P. imbricata	Stuttgart	tro	spike	5.0	reddish	yellow	yellow	reddish bracts	✓	✓	✓	✓

P. integrifolia	Berlin	tro	raceme	4.0	greenish	reddish	reddish	reddish sepals & petals	✓	✓	✓	-
P. jimenezii	Heidelberg	tro	raceme	5.0	greenish	reddish	reddish	reddish sepals & petals	✓	-	✓	-
P. longissimiflora	Berlin	chi	raceme	9.5	greenish	greenish	white	greenish/white sepals & petals	✓	✓	✓	✓
P. maidifolia	Stuttgart	tro	spike	5.0	reddish	white	white	reddish bracts	✓	✓	✓	✓
P. nigra var. nigra	Berlin	tro	spike	8.0	reddish	reddish	reddish	reddish sepals & petals	✓	✓	✓	-
P. oliva-estevae	Berlin	tro	spike	5.0	reddish	reddish	white	reddish bracts	✓	✓	✓	✓
P. orchidifolia	Berlin	tro	raceme	4.5	greenish	reddish	reddish	reddish sepals & petals	✓	-	✓	-
P. poeppigiana	Berlin	tro	spike	7.0	greenish	reddish	reddish	reddish sepals & petals	✓	✓	✓	✓
P. recurvata	Berlin	chi	spike	7.0	greenish	greenish	white	greenish/white sepals & petals	✓	✓	✓	-
P. rubronigriflora	Berlin	tro	raceme	4.5	reddish	reddish	reddish	reddish sepals & petals	✓	-	✓	-
P. sceptrigera	Berlin	tro	spike	6.5	greenish	yellow	yellow	yellow/white sepals& petals	✓	✓	✓	✓
P. spicata	Heidelberg	tro	spike	5.0	reddish	reddish	reddish	reddish sepals & petals	✓	-	✓	-
P. sprucei	Berlin	tro	raceme	5.5	reddish	reddish	reddish	reddish sepals & petals	✓	-	✓	-
P. suaveolens	Berlin	tro	raceme	5.0	greenish	yellow	white	yellow/white sepals& petals	✓	-	✓	-
P. utcubambensis	Berlin	tro	panicle	4.0	reddish	reddish	reddish	reddish sepals & petals	✓	-	✓	-
P. wendlandii	Berlin	tro	spike	5.5	reddish	yellow	yellow	reddish bracts	✓	✓	✓	✓

tro = trochilophilous; chi = chiropterophilous

Supplementary Table S2: Results of the PERMANOVA and PERMDISP of three component groups: (A) amino acids, (B) sugars and inorganic ions, (C) Alanine. Data on leaves, nectaries, and nectar of four *Pitcairnia* species with red flowers or bracts and four *Pitcairnia* species with yellow/white or greenish/white flowers were used for the calculations. The significance level (*) for both methods is p ≤ 0.001.

	Degrees of Freedom (df)	Pseudo-F (F)	R^2	PERMANOVA *p*-value	PERMDISP *p*-value
**(A) Amino acids**					
Flower colour	1	29.95	0.58	0.001 *	0.001
Residuals	22		0.42		
Total	23		1.00		
**(B) Sugars, inorganic ions**					
Flower colour	1	7.68	0.26	0.001 *	0.007
Residuals	22		0.74		
Total	23		1.00		
**(C) Alanine**					
Flower colour	1	97.54	0.82	0.001 *	0.038
Residuals	22		0.18		
Total	23		1.00		

Supplementary Table S3: Ratio of different compounds in leaves, nectaries and nectar of *Pitcairnia* species separated by flower colour.

Flower colour	Tissue	Reddish sepals & petals	Reddish bracts	Yellow/white sepals & petals	Greenish/white sepals & petals
Sugars/amino acids	Leaf	5.4 ± 4.0	4.6 ± 2.9	6.8 ± 7.5	7.0 ± 1.7
	Nectaries	5.9 ± 2.9	5.0 ± 3.6	3.8 ± 1.1	7.5 ± 1.8
	Nectar	81.7 ± 112.7	244.1 ± 351.6	1076.8 ± 1018.0	759.8 ± 222.4
Sugars/inorg. ions	Leaf	0.1 ± 0.1	0.1 ± 0.1	0.1 ± 0.1	0.4 ± 0.4
	Nectaries	1.5 ± 1.0	1.2 ± 0.8	1.2 ± 0.8	1.9 ± 0.6
	Nectar	166.3 ± 141.0	213.3 ± 161.1	99.5 ± 65.6	80.0 ± 38.7
