# Supplementary figures and images for: Origin and Function of Amino Acids in Nectar and Nectaries of Pitcairnia Species with Particular Emphasis on Alanine and Glutamine
Source: Plants (Basel). 2023 Dec 20;13(1):23. doi: 10.3390/plants13010023 (PMC10780904; doi:10.3390/plants13010023)

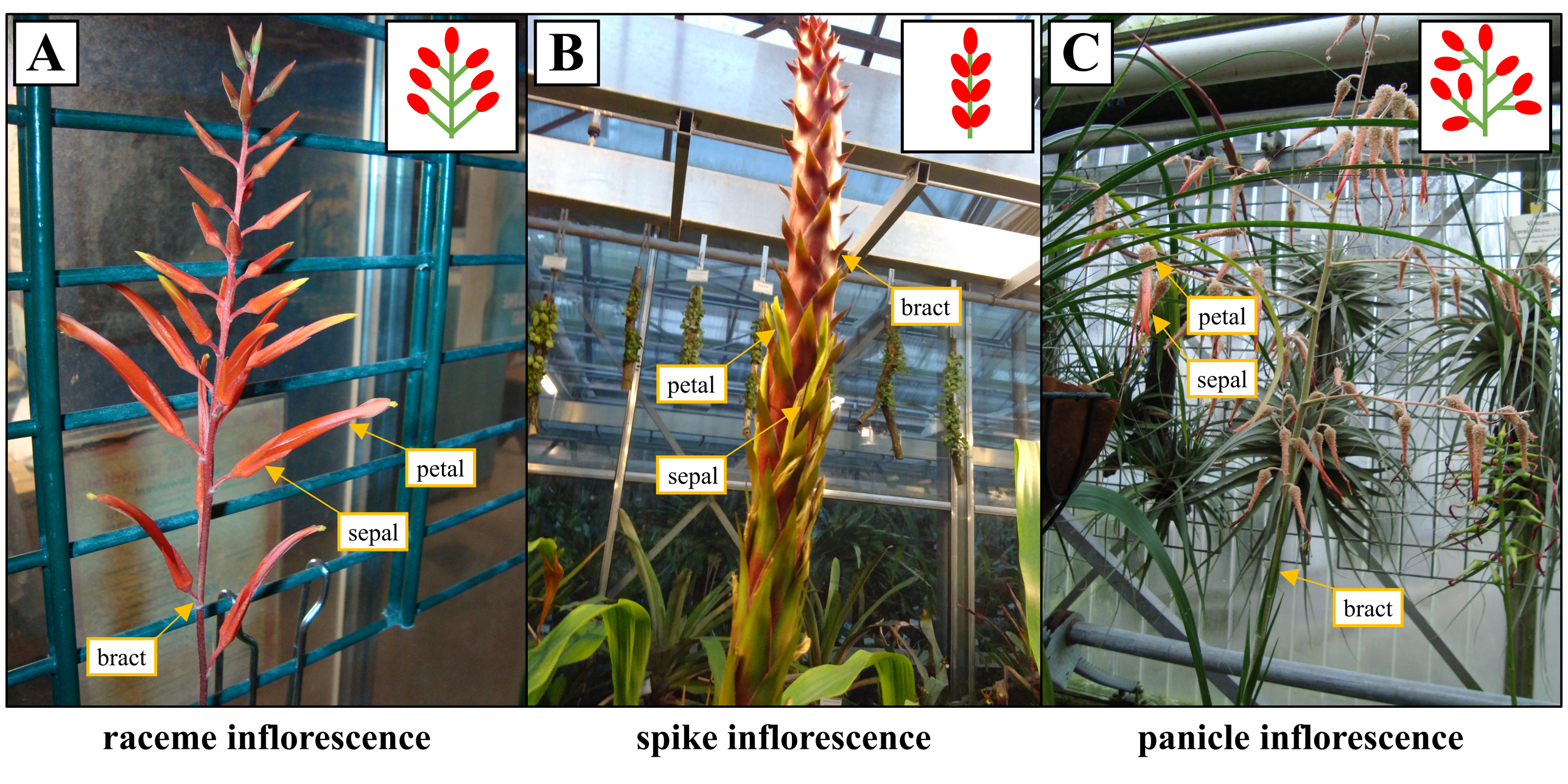

Supplement: Supplementary file 1 [file plants-13-00023-s001.zip › Supplementary_Figure_S1.tif]

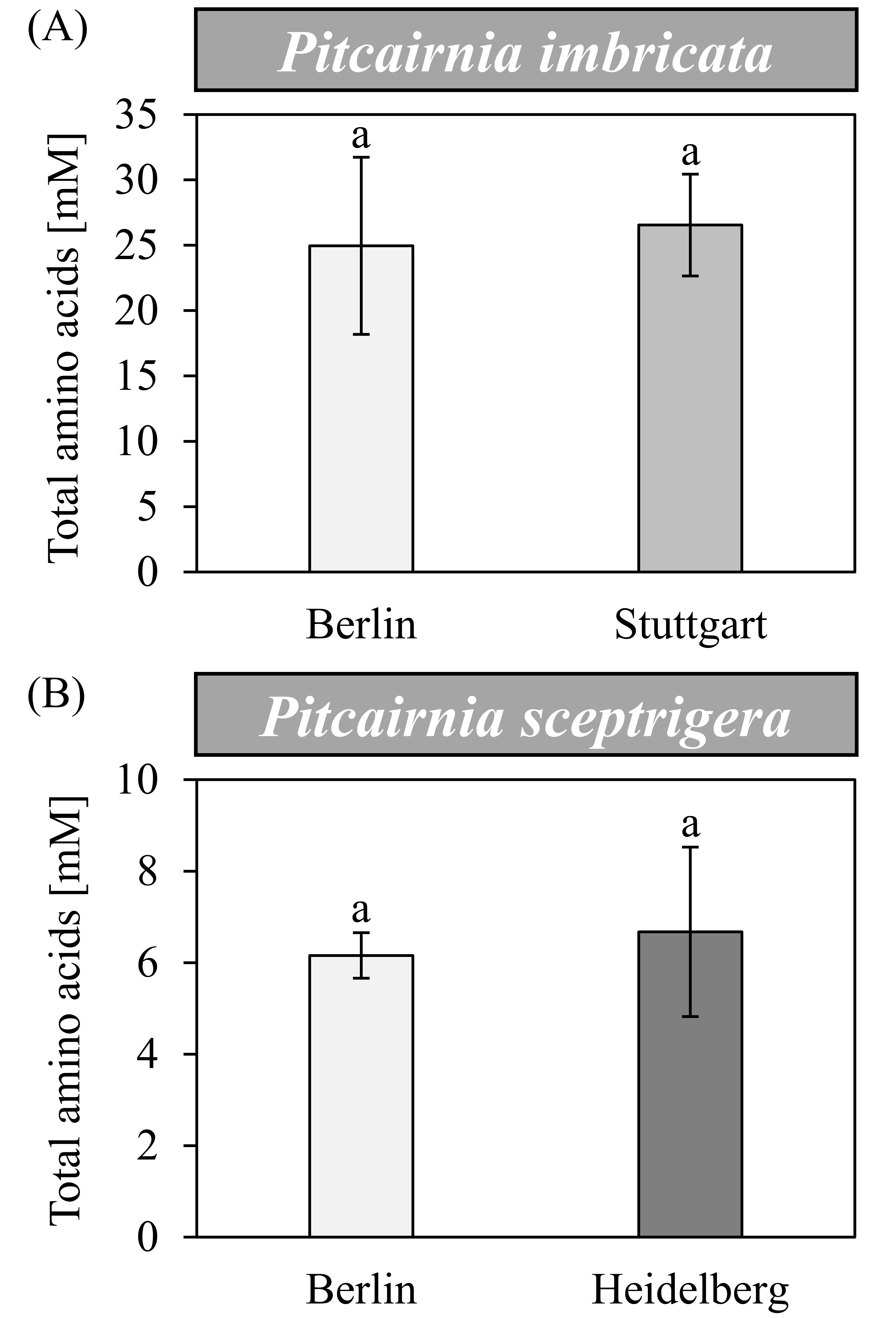

Supplement: Supplementary file 1 [file plants-13-00023-s001.zip › Supplementary_Figure_S2.tif]
